# Developing Hospital at Home tariffs in Denmark: a time-driven activity-based microcosting approach within a randomised controlled trial

**DOI:** 10.1136/bmjopen-2025-113738

**Published:** 2026-04-20

**Authors:** Bettina Wulff Risør, Iben Duvald, Camilla Palmhøj Nielsen, Nasrin Tayyari

**Affiliations:** 1 DEFACTUM, Central Denmark Region Center for Public Health and Quality Improvement, Aarhus, Denmark; 2 Interdisciplinary Center for Organizational Architecture (ICOA), Department of Management, Aarhus University, Aarhus, Central Denmark Region, Denmark; 3 The Emergency Department, Viborg Regional Hospital, Regional Hospital Central Jutland, Viborg, Denmark; 4 Aarhus University Department of Public Health, Aarhus, Central Denmark Region, Denmark

**Keywords:** HEALTH ECONOMICS, Health Care Costs, Health policy, Health Services, Health economics, Emergency Service, Hospital

## Abstract

**Objectives:**

To develop an empirically grounded, activity-based tariff framework for Hospital at Home (HaH) services using time-driven activity-based costing (TDABC) and micro-costing to support transparent and equitable reimbursement for acute elderly care delivered at home.

**Design:**

Microcosting study embedded within a randomised controlled trial (RCT) comparing HaH with conventional hospital admission in Denmark.

**Setting:**

Three municipalities in the Central Denmark Region in collaboration with emergency department physicians at a regional hospital.

**Participants:**

A consecutive subsample of 107 elderly acute patients enrolled in the RCT between June 2022 and February 2024. Resource use for HaH activities was measured prospectively using microcosting logs, time-motion observations and administrative records.

**Main outcome measures:**

Empirically derived tariffs per HaH visit (first and subsequent) calculated using an eight-step TDABC framework incorporating process mapping, resource identification, capacity cost rates and time equations. Sensitivity analyses tested robustness to variation in key cost drivers.

**Results:**

The mean total tariff was €338.89 (95% CI €310.94 to €351.49) for first visits and €207.81 (95% CI €200.70 to €215.69) for subsequent visits, including treatment and transport components. Staff time was the principal cost driver, while equipment, overhead and travel reimbursement had smaller effects. The framework accommodates variation in staffing, geography and visit intensity and can be used to estimate total costs across diverse HaH pathways.

**Conclusions:**

A transparent and reproducible tariff-development framework for HaH services was established using TDABC and microcosting. The model aligns reimbursement with actual resource use and care complexity and provides a transferable template for economic evaluation and operational planning.

**Trial registration number:**

NCT05360914.

**STRENGTHS AND LIMITATIONS OF THIS STUDY**

- Prospective microcosting embedded within a randomised controlled trial enabled activity-level measurement of staff time and resource use.
- The eight-step time-driven activity-based costing framework was applied systematically, including process mapping, capacity cost rate calculation and development of time equations.
- Activities and staff time were recorded prospectively using structured microcosting logs, and costs were assigned using national salary rates and equipment cost estimates.
- Transportation time and costs were modelled explicitly as a separate component based on observed travel patterns.
- Medication costs and broader care-related activities were excluded from the tariff calculations, which may limit completeness for full episode costing.

## Introduction

 Health systems worldwide face increasing pressure to balance rising demand for high-quality care with the need to control costs.^1^ Demographic change, the growing burden of chronic disease and constrained resources have driven policymakers and providers to develop innovative models of service delivery that improve patient outcomes without escalating expenditure.^2^ Hospital at Home (HaH) is one such model, providing acute treatment in patients’ own homes as a substitute for conventional inpatient admission. HaH has been associated with reductions in mortality, hospital readmissions and healthcare resource use compared with traditional hospital care, while also demonstrating high levels of patient satisfaction and feasibility across diverse healthcare contexts.^3^,^5^

Despite growing evidence for the clinical and patient-centred benefits of HaH, the economic implications of this approach remain less clearly defined.^6^ Delivering HaH introduces resource requirements and logistical complexities distinct from standard hospital admission, including workforce planning, equipment provision, digital technology and transport.^7^ The organisation and cost structure of HaH is therefore both multifaceted and context-dependent, with the potential for significant variation across settings and diagnoses.^8^ Establishing a robust understanding of these cost drivers is critical for informing resource allocation decisions, tariff development and the wider adoption of HaH within health systems.^6 9^

Conventional hospital cost estimation methods, which typically aggregate costs at the departmental or service level, are inadequate for evaluating decentralised and cross-sectoral patient-specific models such as HaH.^6 9^ Traditional methods often obscure the true costs of care processes and do not capture resource consumption at the level of individual patients or pathways. As a result, there is limited transparency regarding the actual costs incurred in providing care at home, impeding effective commissioning, reimbursement and service planning.^10 11^

Time-driven activity-based costing (TDABC) has emerged as a leading methodological approach for healthcare cost analysis, particularly in the context of value-based healthcare and innovative service models.^12^,^14^ TDABC allocates costs by directly mapping the resources and time expended on each activity within a care pathway, thereby enabling more accurate, granular and reproducible cost measurement.^9 12^ The TDABC framework has been successfully applied to a range of healthcare interventions, including chronic disease management and integrated multidisciplinary care, and is increasingly recommended for costing new models of service delivery.^15^,^19^ TDABC facilitates the identification of cost variation, supports process improvement and generates information essential for value-based purchasing and payment models.^20^

Notwithstanding these advantages, the application of TDABC to HaH is still evolving, with methodological challenges relating to data availability, process mapping and the attribution of shared or indirect costs.^21^ Nevertheless, international experience demonstrates that TDABC can inform the development of activity-based tariffs for home-based care, offering a means to align payment mechanisms with actual resource use and care complexity.^15 16^

In this study, we apply a TDABC via a microcosting approach to the HaH model in Danish healthcare. Our objective is to establish empirically grounded activity-based tariffs for cross-sectoral HaH services by systematically quantifying resource use, process times and associated costs across the HaH care pathway. The study aims to advance the evidence base for HaH costing and to support transparent and reproducible tariff-setting within integrated healthcare systems.

## Methods

### Study design

This study was embedded within an ongoing randomised controlled trial (RCT) evaluating HaH compared with standard hospital admission for elderly acute medical patients in the Central Denmark Region (ClinicalTrials.gov NCT05360914). The microcosting data used for tariff development were collected prospectively between June 2022 and February 2024. The trial is conducted in collaboration between three municipalities, general practitioners (GPs), the regional emergency department (ED) and the prehospital service.

Patients aged 65 years and above with an acute but stable medical condition, such as pneumonia, erysipelas or urinary tract infection, are screened jointly by the referring GP or emergency physician and the ED physician. Eligible patients living in one of the participating municipalities are randomised in a 1:2 ratio to receive either standard hospital admission or HaH treatment at home delivered by municipal acute-care nurses under the clinical responsibility of an ED physician. The primary outcomes are 30-day acute readmission and health-related quality of life, while secondary outcomes include functional mobility, mortality, healthcare resource use and costs. Full details of the protocol are published elsewhere.^22^

As part of the RCT, a microcosting study was conducted to systematically capture all activities and time expenditure associated with the delivery of HaH services. A purpose-designed microcosting log was developed to record detailed information on resource use, including the specific activities performed and the time spent by each healthcare professional involved in HaH care. The full methodology and feasibility of the developed microcosting log are described in detail elsewhere.^23^ In the present analysis, data derived from the microcosting logs form the empirical basis for tariff development.

### Setting

The study was conducted in three municipalities within the Central Denmark Region. The Danish healthcare system is publicly funded and organised across three administrative levels. The state provides regulation and overall financing, the 5 regions are responsible for hospitals and general practice and the 98 municipalities deliver home nursing, rehabilitation and long-term care.

In recent years, Danish municipalities have established acute teams staffed by nurses who are specialised in providing acute treatment and selected hospital-level procedures in patients’ homes. These municipal acute nurses are supported by municipal home nurses, who perform delegated nursing tasks such as medication administration, wound care and patient monitoring. Municipal care staff provide personal care and practical assistance when required, ensuring continuity between acute and long-term municipal services.

HaH in this study was delivered by municipal acute nurses in close collaboration with GPs, ED physicians, municipal home nurses and care staff. The municipal acute nurses provided 24-hour in-home clinical assessment and treatment under the supervision of ED physicians. The ED physician retained clinical responsibility for patient treatment, supervised the acute nurses through daily virtual ward rounds and provided medical consultation when needed.

### Participants

The microcosting study included a consecutive sample of 107 elderly acute patients treated within the HaH arm of the RCT. No additional inclusion or exclusion criteria were applied for the microcosting study beyond those defined in the RCT protocol. Patients lived either in their own homes or in nursing homes within the participating municipalities.

A treatment episode was defined as the full HaH course from admission to formal termination of the HaH treatment, after which responsibility reverted to usual primary or municipal care services. Episodes were also considered terminated if the patient required escalation of care and was converted to conventional hospital admission, at which point the HaH episode ended.

### Data sources and microcosting

Activity-level resource use during each HaH episode was recorded prospectively using specifically developed microcosting logs. For every visit, staff registered activity type, duration, professional category and time spent on treatment, communication, documentation and transport. Supplementary administrative data provided information on staff salaries, equipment and overheads. These data constituted the empirical foundation for the TDABC analysis and tariff development.

Medication costs were excluded because they vary substantially between diagnoses and treatment regimens. In the tariff development, only the resource use of treating staff (acute nurses, municipal home nurses and physicians) was included, while activities undertaken by municipal care staff were not regarded as part of the treatment component.

### Development of tariffs using TDABC

**Figure 1:**
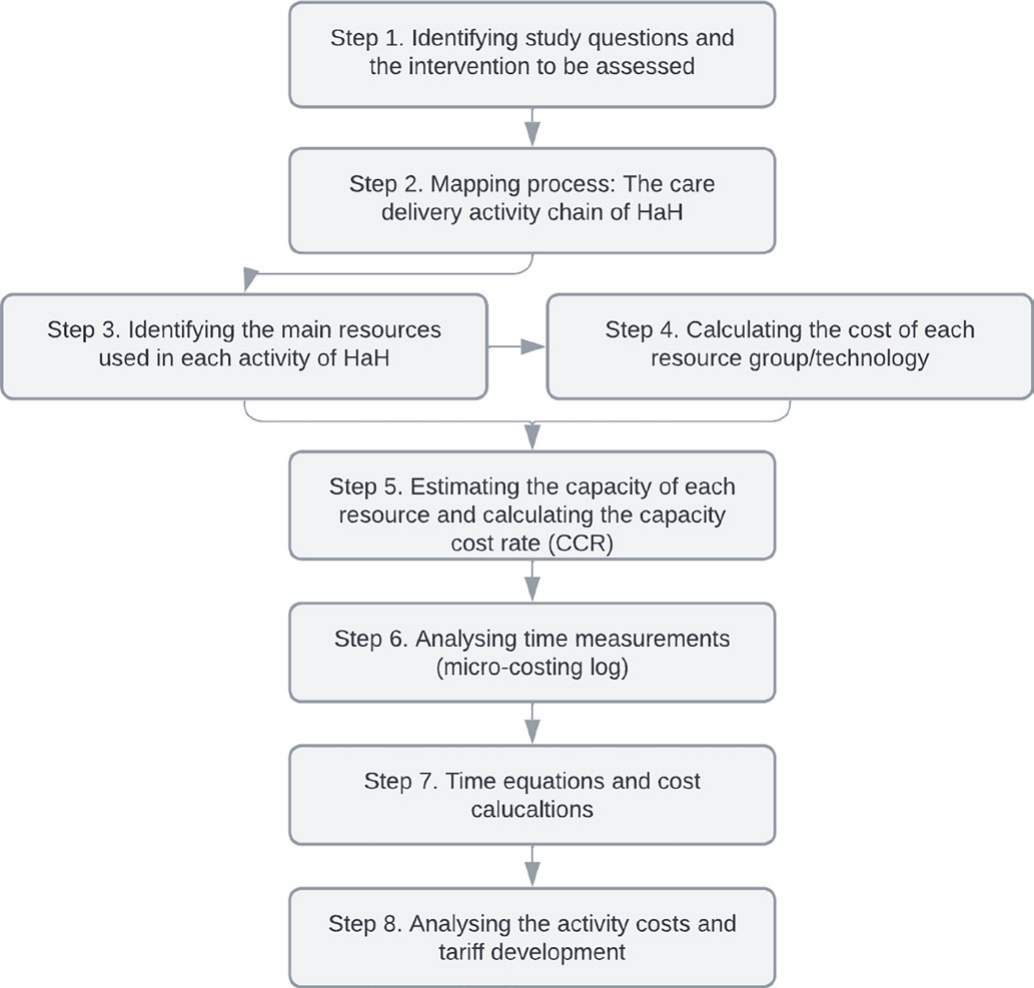
The eight-step process in the applied time-driven activity-based costing (TDABC) framework. HaH, Hospital at Home.

We developed tariffs for HaH services using the eight-step TDABC framework, adapted to capture the specific processes and resources inherent in this model.^12 24^ The process is summarised in figure 1, which provides an overview of the stepwise methodology. A brief description of each stage is presented here, and a detailed description of each stage is provided in online supplemental file 1.

#### Step 1: Identification of study question and technologies

The primary aim was to establish an activity-based tariff system for HaH services, focusing specifically on the resource and cost components associated with home-based treatment, in contrast to standard hospitalisation.

#### Step 2: Process mapping; the care-delivery value chain

**Figure 2:**
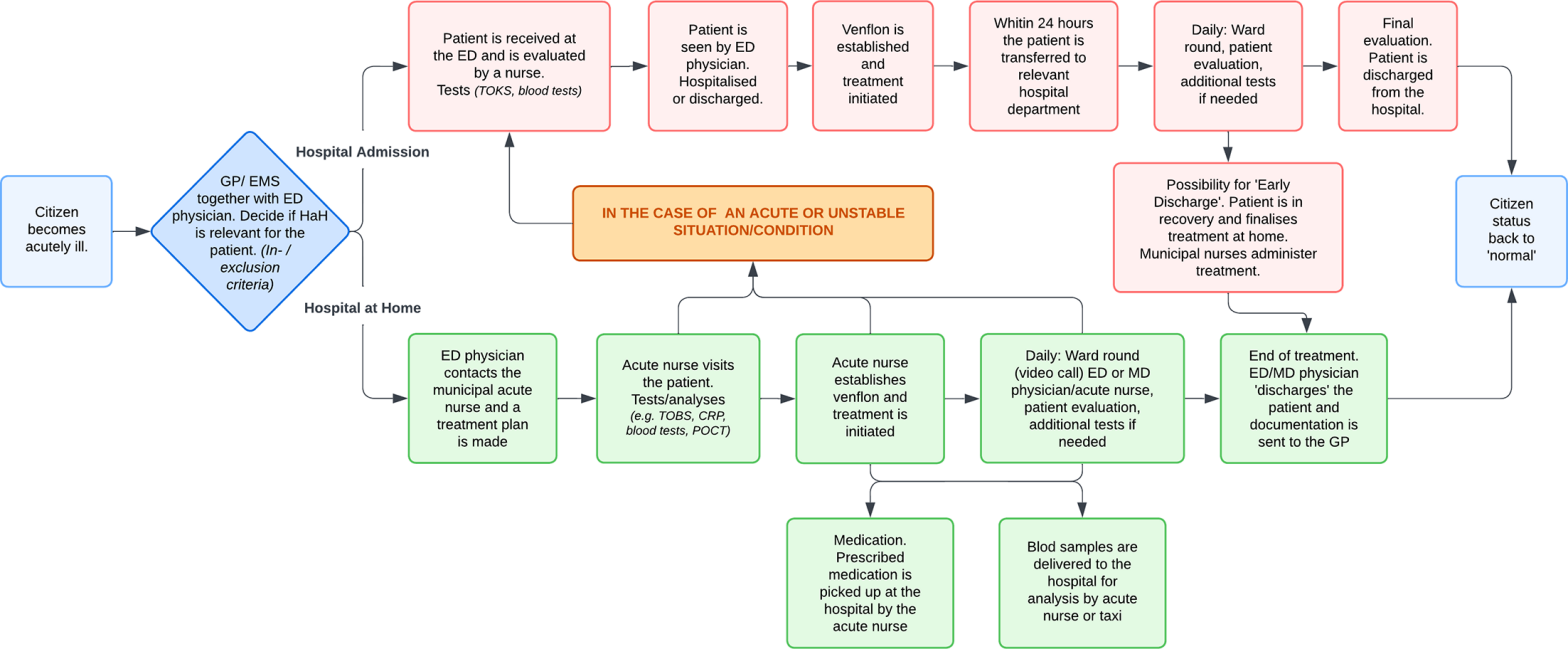
Process map showing activities in the HaH model and standard hospitalisation. ED, emergency department; GP, general practitioner; HaH, Hospital at Home; CRP, C-reactive protein; ED, Emergency department; EMS, Emergency medical services; MD, Medical department; POCT, Point-of-care testing; TOBS, Triage and observation of vital signs; TOKS, Treage and early warning score.

We constructed a comprehensive process map detailing clinical and logistical activities in HaH care vs standard hospitalisation, enabling visual analysis of resource use across the treatment pathway (figure 2).

#### Step 3: Identification of resources used in HaH activities

We conducted a detailed resource inventory for each activity identified in the process map. Resource use was categorised into labour (eg, acute nurse, home nurse, ED physician), equipment (eg, portable diagnostic devices, utensils) and indirect costs (overhead). Medication costs were not included in the analysis as these vary significantly depending on the patients’ diagnosis and condition.

#### Step 4: Measurement of resource use for each resource group

Resource use was measured for each care activity by recording healthcare staff time across categories, including clinical care, documentation, communication and transport. Transportation time covered travel to/from patients’ homes and transfer of samples and medications. Data were collected via microcosting logs, structured interviews and administrative records. Equipment use was documented through staff interviews and verified using local systems.

#### Step 5: Calculation of capacity cost rate (CCR)

*Staff costs:* For each staff group, an hourly rate was calculated based on annual salary and 1122 effective hours/year, following Danish Medicines Council guidelines.^25^ Overhead was added at 18% for regional staff^26^ and 10% for municipal staff.^27^ The capacity cost rate (CCR) was derived by dividing total annual cost (salary plus overhead) by practical capacity, ie, hours available for direct clinical work after adjustments (online supplemental file 2). The HaH service was delivered within existing municipal and hospital emergency structures, and no additional standby capacity was established specifically for the study. On-call availability was therefore considered already incorporated into the salary-based CCRs, rather than costed as a separate standby resource.

*Equipment costs:* Equipment costs included purchase, depreciation, maintenance and consumables, with data from clinical engineering departments and staff. Annualised investment costs were calculated using standard annuitisation based on expected useful life. CCR was computed by dividing annualised cost by estimated operational hours, in line with international health economic evaluation guidelines^28^ (online supplemental file 3).

#### Step 6: Analysing time measurements

For each HaH activity, key time drivers were identified and quantified using microcosting logs maintained by municipal staff for each patient visit. Logs included staff category, visit duration, activity content and time spent on communication, documentation and transport (data are presented in online supplemental file 4). Supplementary data on equipment use and specific activities were collected through structured interviews with healthcare professionals.

#### Step 7: Development of time equations

Activity-specific time equations were developed to estimate resource use per HaH component. For each activity (eg, patient care, communication, documentation, transport, equipment), mean time per visit by staff category and the proportion of visits per group were derived from microcosting logs. These were combined with CCRs to construct cost equations per activity and staff type. All equations were validated through administrative data and staff interviews for consistency and transparency.

#### Step 8: Total cost calculation and tariff development

The validated time equations and CCRs were aggregated to estimate total cost per HaH visit and inform tariff structure. This calculation included direct patient treatment, communication, documentation and additional clinical activities, as well as equipment.

Tariffs were stratified by visit type (first vs following) and nine time periods (weekday, Saturday, Sunday/holiday; each split into day, evening, night).

For first visits, which were exclusively conducted by acute nurses, treatment cost within each time period was calculated by multiplying the observed mean time use for all involved staff categories by their respective CCR. This included time spent on the home visit itself, documentation, communication and time incurred by other healthcare professionals involved in the episode. Activity-based costs were added as the mean frequency of each activity per visit multiplied by its corresponding CCR.

For following visits, which could be conducted by either municipal home nurses or acute nurses, total resource use per visit was first estimated separately for each visit type within each time period. For each visit type, all time components, including time spent by other involved staff categories, were multiplied by their respective CCRs to derive a total cost per visit type. A weighted mean treatment cost was then calculated based on the observed relative proportion of acute nurse and home nurse visits within the given time period. Activity-based costs were incorporated in the same manner as for first visits. An example calculation is available in online supplemental file 5.

The resulting treatment tariff for each combination of time period and visit type was therefore calculated as follows:


$$\begin{array}{l} Tariff_{\left(treatment,p,v\right)}=\sum_{n}w_{\left(n,p,v\right)}\left[\sum_{r}\left(T_{\left(visit,r,p,v,n\right)}\;\;+T_{\left(comm,init,r,p,v,n\right)}+T_{\left(comm,rec,r,p,v,n\right)}\;\;+T_{\left(doc,r,p,v,n\right)}\right)\times CCR_{\left(salary,r\right)}\right]+\sum_{a}\left(N_{\left(a,p,v\right)}\times CCR_{a}\right) \end{array}$$

where:

- *p* = time period (one of nine categories: weekday, Saturday or Sunday/holiday; each subdivided into day, evening or night).
- *v* = visit type (first visit or following visit).
- *n* = visit provider type (acute-led or home-led visit); for first visits, n = acute.
- *w*_(n,p,v__)_ = observed proportion of visits delivered by provider type n within time period p and visit type v; for first visits, *w*_₍acute,p,first₎_ = 1.
- *r* = staff category involved in the visit episode (eg, acute nurse, home nurse, hospital nurse, emergency/medical physician).
- *T*_₍visit,r,p,v,n₎_ = mean time spent by staff category *r* on direct patient treatment and preparation for visits of type *v* delivered by provider type *n* in time period *p.*
- *T*₍_comm,init,r,p,v,n_₎ = mean time spent by staff category *r* initiating communication (eg, contacting other healthcare professionals).
- *T*₍_comm,rec,r,p,v,n_₎ = mean time spent by staff category *r* receiving and responding to communication.
- *T*₍_doc,r,p,v,n_₎ = mean time spent by staff category *r* on documentation after the visit.
- *CCR*₍_salary,r_₎ = capacity cost rate for staff category *r*, including salary and overhead.
- *a* = clinical activity (eg, Point-of-care testing (POCT), C-reactive protein test (CRP), Triage and observation of vital signs (TOBS), venflon, intravenous treatment).
- *N*₍_a,p,v_₎ = mean frequency of activity *a* per visit within time period *p* and visit type *v.*
- *CCR*_a_ = capacity cost rate (unit cost) associated with activity *a*, including equipment and consumables as applicable.

Transport cost combined staff transport time, valued at wage rates and weighted by staff proportions and travel distance, based on driving time at 40 km per hour and reimbursed at the official low rate. Transport was estimated separately for each municipality and expressed as a mean cost per visit. To enhance transparency and applicability across settings, transportation was reported separately from the treatment component of the tariff.


$$\begin{array}{l} C_{\left(transport,p\right)}=\sum_{n}w_{\left(n,p\right)}\left(T_{\left(trans,n,p\right)}\times CCR_{\left(salary,n\right)}\right)\\ \;+\left(D_{\left(mean,p\right)}\times R_{\left(travel\right)}\right) \end{array}$$

where:

- *p* = time period (one of nine categories: weekday, Saturday or Sunday/holiday×day, evening or night).
- *n* = visit provider type (acute-led or home-led visit).
- *w_(n,p)_* = observed proportion of transport performed by provider type *n* within time period *p.*
- *T*₍_trans,n,p_₎ = mean transport time per visit for provider type *n* in time period *p.*
- *CCR*₍_salary,n_₎ = capacity cost rate for staff salary (including overhead) for provider type *n.*
- *D*₍_mean,p_₎ = estimated mean driving distance per visit in time period *p*, calculated from observed transport time and an assumed average speed of 40 km/hour.
- *R*₍_travel_₎ = government set mileage reimbursement rate (low tariff).

Final treatment tariffs for first and following visits were calculated by aggregating cost estimates across all time periods using weighted means based on the observed visit distribution. Transport costs were summarised similarly, weighted across time periods and municipalities. The final tariffs combined treatment and transport components, resulting in two overall tariffs: one for first visits and one for following visits, each reflecting the total expected cost per visit.

### Uncertainty analysis

To quantify sampling uncertainty in the tariff estimates, we conducted a probabilistic uncertainty analysis using Monte Carlo simulation. The tariffs are deterministic functions of empirically observed mean resource-use inputs (time use and activity frequencies) combined with fixed unit-cost parameters. Uncertainty therefore arises from sampling variability in the observed resource-use inputs rather than from model estimation.

For each time component and activity frequency included in the staff time and equipment components of the tariff model, we derived the mean and corresponding SE from the underlying dataset. In 10 000 Monte Carlo iterations, these inputs were sampled from normal distributions centred on the observed means with SD equal to their empirical SEs. Simulated values were truncated at zero to avoid implausible negative time or activity inputs. In each iteration, the full deterministic tariff model was recalculated, generating empirical distributions for the staff time component, equipment component, total tariff for first visits, total tariff for following visits and the transport tariff.

Percentile-based 95% CIs were obtained from the 2.5th and 97.5th percentiles of the simulated distributions. Unit-cost parameters, including CCRs, overhead assumptions and mileage rates, were held constant in the probabilistic analysis, as these represent parameter assumptions rather than sampling variability in observed resource use.

### Sensitivity analyses

Deterministic one-way sensitivity analyses were performed to examine the robustness of the tariff estimates to variation in key cost parameters. Staff, equipment and transport cost parameters were varied by ±10% and ±20% around base-case values. Overhead rates were adjusted to reflect either 0% for both regional and municipal staff, or alternatively 15% for municipal and 25% for regional staff. In these scenarios, the alternative overhead rates replaced the base-case assumptions described in step 5 and were applied through recalculation of the staff CCRs, while practical capacity and all other model inputs were held constant. Travel costs were varied by excluding the mileage rate or increasing it to assess its influence on total tariffs.

### Patient and public involvement

Qualitative interviews undertaken during the pilot phase of the RCT informed the design of the HaH pathway and the refinement of the microcosting procedures applied in this study. However, patients and members of the public were not directly involved in the design or conduct of the tariff-development study.

## Results

A total of 1125 HaH visits were provided to 107 patients, with a mean age of 79 years (SD 8.5). The median number of total visits per patient was 9 (range 2–27), indicating variation in visit intensity across patients. Table 1 summarises characteristics of study population.

**Table 1:** Population characteristics

Age (mean, SD)	79	8.5
Gender (n, %)		
Female	43	40%
Male	64	60%
Municipality (n, %)		
Viborg	47	44%
Silkeborg	33	31%
Skive	27	25%
Resident location (n, %)		
Own home	80	75%
Nursing home	27	25%
Bed days, mean (SD); median (range)	3.8 (3.3)	3 (0–13)
Visits by acute nurse, mean (SD); median (range)	8.8 (5.2)	5 (2–22)
Visits by home nurse, mean (SD); median (range)	1.7 (2.8)	0 (0–10)
Total visits per patient, mean (SD); median (range)	10.5 (6.3)	9 (2–27)
Diagnosis		
Pneumonia	38	36%
Acute cellulitis	24	22%
Urine tract infection	20	19%
Other diagnoses	25	23%

For continuous variables, values are presented as mean (SD) and median (range).

To ensure data confidentiality, the reported range is calculated as the mean of the five lowest and five highest observed values rather than the absolute minimum and maximum.

### Treatment component of the tariff

The mean weighted treatment cost per first visit was €234.57 (95% CI €207.43 to €245.35), based on 107 observations (table 2). First visits most frequently occurred on weekday evenings and days, which also contributed the most to the overall mean due to their high weights. Weekend and night visits were less common, and although these categories sometimes had higher unit costs, their overall influence was limited. The staff time component amounted to €216.70 (95% CI €189.48 to €227.21) and the equipment component to €17.87 (95% CI €16.35 to €19.56).

**Table 2:** Treatment cost per visit (2025 €)

First visit							
	n	Weight	Cost per visit		Weighted component		**Total cost (weighted)**
			Staff time	Activities	Staff time	Activities	
Weekday							
Day	35	0.327	205.76	19.88	67.30	6.50	73.81
Evening	53	0.495	212.00	17.23	105.01	8.53	113.54
Night	0						
Saturday							
Day	7	0.065	148.21	8.62	9.70	0.56	10.26
Evening	2	0.019	261.60	12.62	4.89	0.24	5.13
Night	2	0.019	399.99	32.86	7.48	0.61	8.09
Sunday							
Day	2	0.019	298.59	14.17	5.58	0.26	5.85
Evening	6	0.056	298.58	20.66	16.74	1.16	17.90
Night	0						
Total	107	1.00			**216.70**	**17.87**	**234.57**
95% CI					(189.48 to 227.21)	(16.35 to 19.56)	(207.43 to 245.35)

Following visits							
	n	Weight	Cost per visit		Weighted component		Total cost (weighted)
			Staff time	Activities	Staff time	Activities	
Weekday							
Day	412	0.405	96.59	9.67	39.09	3.91	43.00
Evening	254	0.250	81.34	4.87	20.30	1.21	21.51
Night	72	0.071	83.81	4.53	5.93	0.32	6.25
Saturday							
Day	78	0.077	108.83	8.02	8.34	0.61	8.95
Evening	44	0.043	105.02	4.36	4.54	0.19	4.73
Night	12	0.012	118.64	4.88	1.40	0.06	1.46
Sunday							
Day	86	0.084	114.42	7.20	9.67	0.61	10.27
Evening	45	0.044	112.86	4.19	4.99	0.19	5.17
Night	15	0.015	130.39	14.81	1.92	0.22	2.14
Total	1018	1.00			**96.17**	**7.32**	**103.49**
95% CI					(93.03 to 99.24)	(6.41 to 7.78)	(100.05 to 106.37)

Proportions may not sum to exactly 1.00 due to rounding.

Bold values represent total weighted mean costs across all time periods, calculated using the observed distribution of visits.

For following visits (n=1018), the mean weighted treatment cost per visit was €103.49 (95% CI €100.05 to €106.37). The majority of these visits took place during weekday days and evenings, whereas visits during nights and weekends were less frequent. The staff time component was €96.17 (95% CI €93.03 to €99.24) and the equipment component €7.32 (95% CI €6.41 to €7.78).

Thus, treatment costs were highest during night and weekend periods but had limited effect on the total weighted mean tariff because most visits occurred during regular weekday hours.

### Transport component of the tariff

The estimated transport component of the tariff was based on observed transportation patterns across all municipalities (table 3). Transportation time and distance were recorded for 495 visits, representing the subset of visits where transport was registered. For the remaining visits, transportation was not recorded, which in several cases reflected that no transport was required, for example, when patients resided in nursing homes or in close proximity to the care provider.

**Table 3:** Transport cost per visit (2025 €)

All municipalities				
Mean transportation time (min)	Mean staff transportation cost	Mean travel distance (km)	Mean travel cost	Total transportation cost (staff+travel)
Weighted	Weighted	40 km per hour	Low rate*	Per visit
77	89.52	51	14.81	104.32 (95% CI 97.42 to 112.37)

* Low rate = €0.29/km.

The mean transportation time per visit was 77 min, corresponding to a mean staff transportation cost of €89.52. The average travel distance was 51 km per visit, resulting in a mean travel cost of €14.81 using the low government-set mileage reimbursement rate. The total mean transportation cost, including both staff transportation and travel reimbursement, was €104.32 per visit (95% CI €97.42 to €112.37).

There was some variation in transportation patterns across municipalities and time periods, but the overall weighted means were primarily determined by the distribution of visits in the study population. A detailed breakdown of transport time, staff cost and travel cost by municipality and time period is presented in online supplemental file 6.

### Total tariff per visit

The total tariff for each visit type was calculated as the sum of the treatment component and the transport component. This approach was applied separately for first visits and following visits, resulting in two distinct overall tariffs that reflect the full expected cost per HaH visit. The calculated total tariff was € 338.89 (95% CI €310.94 to €351.49) for first visits and €207.81 (95% CI €200.70 to €215.69) for following visits.

### Sensitivity analyses

The sensitivity analyses revealed that the tariff was primarily driven by staff-related costs, while variations in equipment, overhead and travel rates produced more moderate or minor effects (table 4). Varying the staff time (treatment) component by ±20% resulted in total tariffs ranging from €295.56 to €382.23 for first visits and from €188.55 to €227.00 for following visits, compared with base-case estimates of €338.89 and €207.81, respectively. In contrast, ±20% variation in the equipment component led to comparatively small changes in total tariffs. Adjustments to overhead assumptions and mileage reimbursement rates resulted in moderate changes, with higher overhead rates (15% municipal/25% regional) and a higher mileage rate (€0.50 per km) increasing the total tariffs, while a zero-overhead scenario or exclusion of travel reimbursement reduced them.

**Table 4:** Results from the sensitivity analyses (2025 €)

First visit					
	−20%	−10%	Base case	10%	20%
Cost component					
Staff time (treatment)	295.56	317.23	338.89	360.56	382.23
Equipment	335.32	337.11	338.89	340.68	342.47
Staff time (transportation)	320.99	329.94	338.89	347.85	356.80
Overhead (municipal/regional)			**Base case**		
	**0%/0%**		10%/18%*	15%/25%*	
		317.84	338.89	349.05	
Travel cost rate		**None**	**Base case (low**)	**High**	
	**0**		**€0.29/km**	**€0.5/km**	
		324.09	338.89	349.38	

Following visit					
	−20%	−10%	Base case	10%	20%
Cost component					
Staff time (treatment)	188.55	198.16	207.81	217.38	227.00
Equipment	206.31	207.04	207.81	208.50	209.23
Staff time (transportation)	189.87	198.82	207.81	216.72	225.67
Overhead (municipal/regional)			**Base case**		
	**0%/0%**		10%/18%*	15%/25%*	
		198.40	207.81	212.28	
Travel cost rate		**None**	**Base case (low**)	**High**	
	**0**		**€0.29/km**	**€0.5/km**	
		193.00	207.81	218.26	

* Overhead rates represent percentage supplements applied to salary costs to account for indirect and administrative expenses. Two rates are shown because staff employed in municipal and regional settings are subject to different overhead assumptions. The base-case assumptions were 10% for municipal staff and 18% for regional staff. The high scenario applied 15% and 25%, respectively. The 0%/0% scenario represents a no-overhead assumption for both sectors.

### Application of the tariff framework

The tariff structure developed in this study enables estimation of total costs for a complete home-based care episode by varying the number of visits and length of stay. For example, for a patient with five bed days and three visits per day, this corresponds to one first visit and fourteen following visits:

1 × €338.89 + 14 × €207.81 = €3248 (rounded to the nearest euro).

By systematically applying the separate tariffs for first and following visits, it is possible to generate robust cost estimates for different patterns of home-based care.

For example, a more intensive episode comprising 3 bed days with four visits per day corresponds to 1 first visit and 11 following visits (12 visits in total):

1 × €338.89 + 11 × €207.81 = €2625 (rounded to the nearest euro).

In contrast, a prolonged episode with lower visit intensity, such as 10 bed days and 2 visits per day, corresponds to 1 first visit and 19 following visits (20 visits in total):

1 × €338.89 + 19 × €207.81 = €4287 (rounded to the nearest euro).

These calculations demonstrate how the tariff framework can be used to accommodate a range of clinical scenarios and care intensities. The approach provides a systematic and reproducible method for estimating the cost of home-based acute care, supporting both economic evaluation and operational planning in diverse healthcare and geographical contexts.

## Discussion

### Principal findings

This study derived empirically based activity-level tariffs for HaH within a Danish cross-sectoral setting. The mean total tariff was €338.89 (95% CI €310.94 to €351.49) for first visits and €207.81 (95% CI €200.70 to €215.69) for following visits, reflecting the combined treatment and transportation components. Staff time accounted for the largest share of total costs in both visit types, whereas equipment and overhead constituted smaller proportions. The mean transportation component was €104.32 per visit (95% CI €97.42 to €112.37), indicating that transport represents a substantial share of resource use in decentralised acute care.

Treatment costs varied across time periods, with higher unit costs observed during evenings, nights and weekends. However, their influence on the weighted mean tariffs was limited, as most visits occurred during regular weekday hours. The resulting tariffs therefore reflect the empirically observed distribution of visits across time periods and provider types within the study population.

### Methodological considerations

Interpretation of the reported tariffs requires consideration of the costing frameworks embedded in hospital and municipal services. In Denmark, hospital treatment is reimbursed through diagnosis-related group tariffs derived from hospital cost accounting systems. These tariffs are constructed from aggregated expenditure data and represent average costs per admission rather than empirically observed activity-level resource consumption. In the Danish setting, diagnosis-related group (DRG) and the underlying hospital cost accounting infrastructure are designed primarily for reimbursement and benchmarking, which limits their suitability for detailed patient-level process costing.^29 30^ Importantly, DRG tariffs define the cost object at the level of an admission, whereas the present framework defines the cost object at the level of a visit based on measured activities and time use. The two approaches therefore rest on structurally different measurement logics.

TDABC has been proposed as a complementary approach that links process mapping and time estimates to CCRs, thereby improving transparency and reproducibility of cost estimates across defined care activities.^12^,^14^ Empirical applications in healthcare suggest that TDABC can be more sensitive to variations in resource use across care processes than conventional accounting-based approaches, provided that process definitions and time estimates are specified transparently.^9 17 19^ In decentralised care models such as HaH, where coordination, communication and transportation are integral components of service delivery, activity-level measurement may therefore be particularly relevant. Nevertheless, international applications in integrated care and home-based acute models remain limited, and most published studies have focused on specific episodes or bundled payment contexts rather than full care pathways.^31 32^

The distinction between bottom-up microcosting and top–down gross-costing further illustrates how methodological choices influence precision and comparability.^33 34^ Top–down approaches may approximate mean costs at aggregated levels but are less suited to capturing variation in time use and process intensity within specific treatment pathways.^33^ Malmmose and Lydersen similarly noted that conventional hospital cost accounting systems are not designed to support detailed patient-level process costing and described TDABC as a means of linking time estimates to resource cost rates.^30^ By deriving visit-level costs from prospectively measured time and activity data, the present study establishes a cost object that is conceptually distinct from accounting-based averages embedded in DRG systems.

From the perspective of economic evaluation, a further distinction concerns average versus marginal costs. As outlined by Drummond *et al*, reimbursement tariffs and accounting-based averages do not necessarily correspond to the opportunity costs relevant for decision-making, particularly when a substantial share of costs is fixed in the short term.^28^ DRG tariffs therefore reflect average expenditure allocations and may not represent the costs that can be avoided if inpatient activity is reduced. This distinction is particularly salient in HaH models, where substitution of inpatient admissions does not necessarily imply proportional reductions in hospital expenditure in the short term, given the high fixed-cost structure of hospital services. In contrast, an activity-based framework makes explicit the resource components associated with each defined care activity, thereby improving transparency regarding which elements of care drive total cost.

These methodological differences have direct implications when DRG-based hospital tariffs and activity-level municipal costing are interpreted within the same economic evaluation. Apparent cost differences between inpatient admission and HaH may otherwise reflect structural differences in accounting conventions and cost aggregation rather than differences in actual resource consumption. Cross-sectoral evaluations therefore require explicit specification of costing perspective, treatment of overhead and capital components, and clarity regarding whether cost estimates are derived from aggregated accounting data or activity-level measurement. Without such conceptual alignment, comparisons risk conflating measurement framework with efficiency differences.

### Comparison with existing evidence

The present study contributes to an established body of international evidence evaluating HaH as a substitute for inpatient admission. Systematic reviews and meta-analyses have consistently reported that HaH interventions for selected patient groups produce clinical outcomes comparable to conventional inpatient care, including no significant difference in mortality and, in several analyses, a reduced risk of readmission.^3^,^535^ For patients with chronic conditions presenting to EDs, evidence further indicates that length of treatment may be longer in HaH compared with inpatient admission, while maintaining similar safety profiles.^5^ Similar conclusions regarding safety and readmissions have been reported in earlier syntheses of admission avoidance models.^3^

In contrast to the relative consistency of clinical findings, economic results vary across HaH models and study contexts. Reviews generally report a tendency towards cost savings compared with inpatient care,^36 37^ but the magnitude and direction of estimated differences differ substantially between studies. This variation reflects differences in costing perspective, time horizon and inclusion of cost components.^4 38^ In addition, several studies have highlighted limitations in the robustness and granularity of cost estimates, often due to reliance on aggregate cost data and heterogeneous accounting methods.^5 6^ Methodological concerns include the use of average unit costs for inpatient care and inconsistent handling of cost components across settings, both of which may influence the estimated magnitude of cost savings.^6^

Importantly, most economic evaluations report total episode-level or admission-level costs. Reported savings therefore reflect aggregated pathway-level estimates rather than activity-based unit costs. The tariff developed in the present study represents a structured unit cost per defined HaH visit, intended to support reimbursement and budgeting within a decentralised model. It does not constitute an estimate of total episode cost and should not be interpreted as evidence of cost savings relative to inpatient admission. By specifying resource use at activity level, the tariff framework provides a transparent component for subsequent cross-sectoral economic evaluation, where episode-level comparisons require conceptual and methodological alignment.

### Strengths and limitations of this study

A key methodological strength of this study is its rigorous, stepwise application of TDABC in combination with prospective microcosting, enabling detailed and reproducible estimation of costs at the activity level. Comprehensive process mapping, use of multiple empirical data sources and systematic validation of time and cost inputs contributed to the transparency and robustness of the tariff estimates. The inclusion of sensitivity analyses further strengthened the reliability of the findings, particularly in relation to variations in overhead, transportation and staff costs.

Nevertheless, several limitations should be considered. The tariff estimates are based on a limited population of older patients and reflect resource use associated with treatment for specific diagnoses commonly encountered in this demographic group, such as pneumonia, cellulitis and urinary tract infection. This may limit the generalisability of results to broader or more heterogeneous patient populations. However, as the included conditions are frequent among older adults, the tariff is likely to be appropriate and relevant for use in comparable age groups and clinical settings.

The generalisability of the tariff framework also depends on the organisation of the specific HaH model. Differences in local service configuration, such as whether treatment is delivered by hospital-employed physicians or by municipal acute nurses, will influence staff time, remuneration levels and collaborative practices, thereby affecting total costs. Variation in staffing composition, task delegation and scheduling of visits may therefore lead to differences in tariff levels across settings.

It is important to note that the tariff presented here captures only direct treatment-related costs and does not include medication costs. Consequently, medication expenses must be added if one wishes to estimate the total cost of an episode of care. The exclusion of medication costs reflects the structural scope of the present activity-based tariff framework, in which highly variable and diagnosis-specific inputs are modelled separately from core care processes in order to preserve transparency and comparability. As medication use may vary substantially across clinical conditions and treatment regimens, inclusion within a general visit-level tariff could reduce interpretability across patient groups. Medication costs can therefore be incorporated separately in subsequent economic evaluations.

The tariff was constructed at the visit level. Repeated measurements, such as multiple observations per patient, were therefore not modelled explicitly, as the primary objective was to estimate average resource use and cost per visit across the study population rather than to quantify within-patient variation. Although we did not conduct analyses to assess the impact of repeated measures, uncertainty and robustness were addressed through sensitivity analyses of key cost drivers. Similarly, while differences in resource use across diagnostic groups may be clinically relevant, the present tariff was constructed as a diagnosis-independent activity-based estimate and does not capture diagnosis-specific cost variation.

Finally, the tariff framework is specific to direct treatment activities and does not encompass potential changes in broader care needs, such as municipal care or support activities before, during or after the HaH episode. Potential impacts on care activities outside the treatment context should therefore be investigated separately to provide a more comprehensive understanding of the full consequences of HaH interventions.

Transferability to other settings or populations will require careful calibration and, where necessary, re-mapping of care processes to ensure contextually appropriate estimates. As with prior TDABC studies, limited integration of data across organisational boundaries might pose a challenge for broader implementation and scalability.^11 17^

### Implications for policy and practice

The results have direct implications for policymakers, commissioners and healthcare providers engaged in the development and financing of innovative care models. By providing a transparent, activity-based tariff system for HaH, this study facilitates reimbursement models that can incentivise efficient resource allocation, promote financial sustainability and enable equitable expansion of home-based care.^20 39^

Beyond HaH specifically, the TDABC framework offers a structured approach to measuring and valuing resource use at activity level, which may be applicable to other municipal health and care services characterised by decentralised delivery and staff-intensive processes. In this context, activity-based costing can strengthen transparency in cost structures and improve alignment between allocated budgets and actual resource consumption. As health systems increasingly move towards value-based healthcare, costing approaches such as TDABC may inform tariff-setting by aligning reimbursement more closely with measured resource use, thereby supporting more coherent and sustainable financing mechanisms across sectors.^20 39^

Explicit modelling of transportation as an adaptable element, together with differentiation between first and following visits and time-of-day variation, strengthens the tariff framework’s capacity to reflect differences in demography, population density and organisational configuration.^40^ Broader implementation requires systematic process mapping, clear definition of care activities and periodic updating of CCRs to reflect changes in wage structures and overhead allocation. Applied in this manner, TDABC may function not only as a tariff development tool but also as a management instrument enabling municipalities to monitor cost drivers and maintain financial oversight of expanding decentralised care models.

## Conclusion

This study presents an empirically grounded, activity-based tariff framework for HaH services in the Danish healthcare context, developed through systematic application of TDABC and prospective microcosting. The mean total tariff was €338.89 (95% CI €310.94 to €351.49) for first visits and €207.81 (95% CI €200.70 to €215.69) for following visits, reflecting the combined treatment and transportation components derived from observed resource use. These estimates provide a transparent and reproducible basis for tariff-setting in decentralised acute care.

Staff time constituted the primary cost driver of the total tariff, whereas equipment, overhead and transportation contributed smaller but measurable components. By mapping care processes and linking time estimates to CCRs, the framework allows tariffs to reflect organisational configuration and visit patterns while remaining adaptable to local variation.

Beyond the specific tariff levels, the study demonstrates how activity-based costing can be operationalised within cross-sectoral care models. The framework offers a structured basis for reimbursement, economic evaluation and operational planning, provided that conceptual alignment between costing approaches across sectors is ensured. Future research should validate the tariff framework in diverse healthcare systems and patient populations to assess generalisability and identify context-specific adaptations. Economic evaluations linking detailed activity-level cost data to patient outcomes in controlled study designs are warranted to assess cost-effectiveness and broader system consequences of HaH and related models.

## Supplementary material

10.1136/bmjopen-2025-113738 online supplemental file 1

10.1136/bmjopen-2025-113738 online supplemental file 2

10.1136/bmjopen-2025-113738 online supplemental file 3

10.1136/bmjopen-2025-113738 online supplemental file 4

10.1136/bmjopen-2025-113738 online supplemental file 5

10.1136/bmjopen-2025-113738 online supplemental file 6

## Data Availability

All data relevant to the study are included in the article or uploaded as supplementary information.
