# Supplementary material for: Developing Hospital at Home tariffs in Denmark: a time-driven activity-based microcosting approach within a randomised controlled trial
Source: BMJ Open. 2026 Apr 20;16(4):e113738. doi: 10.1136/bmjopen-2025-113738 (PMC13110545; doi:10.1136/bmjopen-2025-113738)
Supplement: online supplemental file 1 [file bmjopen-16-4-s001.docx]

### **Supplementary File 1. Development of tariffs using TDABC – detailed description**

We developed tariffs for Hospital at Home (HaH) services using the eight-step Time-Driven Activity-Based Costing (TDABC) framework, adapted to capture the specific processes and resources inherent in this model. The process is summarised in Figure 1, which provides an overview of the stepwise methodology, followed by a detailed description of each stage.

Figure 1. The eight-step TDABC framework. Adopted and adjusted from da Silva Etges et al, 2019.


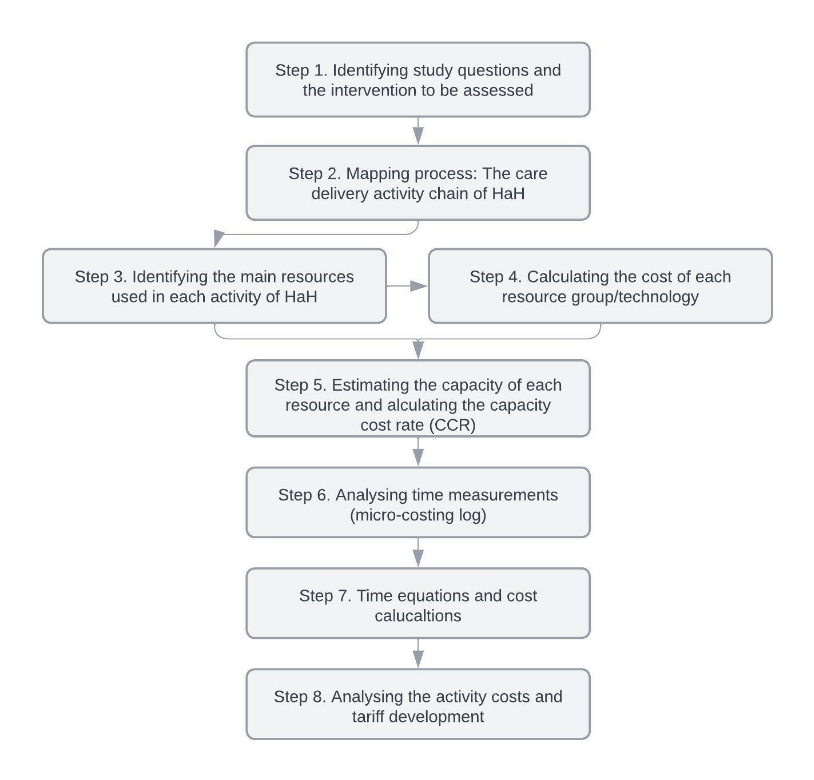


*HaH: Hospital at Home*

#### **Step 1: identification of study question and technologies**

#### The primary aim was to establish **an activity-based tariff system** for HaH services, focusing specifically on the resource and cost components associated with home-based treatment in contrast to conventional hospitalisation.

#### **Step 2: process mapping; the care-delivery value chain**

We constructed a comprehensive process map documenting all activities involved in HaH service delivery relative to standard hospitalisation. This map captured the complete clinical and logistical trajectory, from the onset of acute illness to recovery and termination of treatment. The care-delivery value chain included stages such as home visits, diagnostic services, treatment administration, and patient monitoring. The process map allowed for visualisation and systematic analysis of resource flows throughout the HaH treatment model.

Figure 2, Process map showing activities in the Hospital at Home model and standard hospitalisation


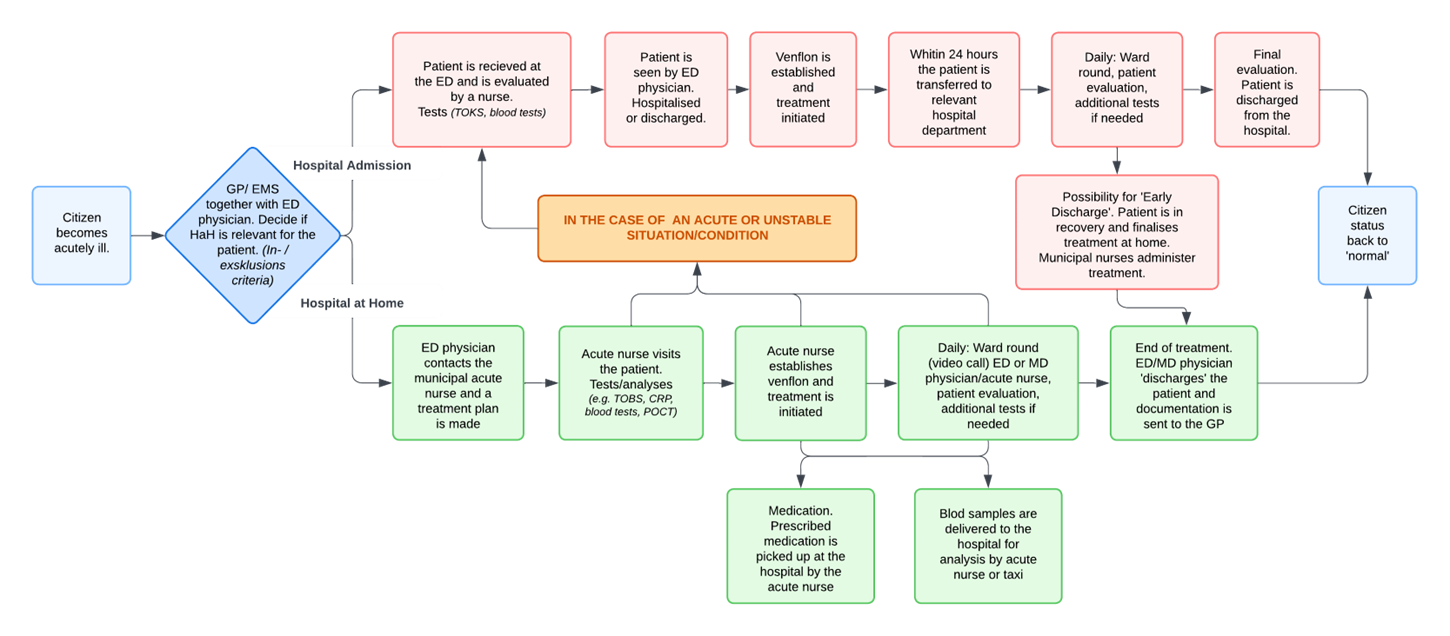


#### **Step 3: Identification of resources used in HaH activities**

We conducted a detailed resource inventory for each activity identified in the process map. Resource use was categorised into labour (e.g., acute nurse, home nurse, ED physician), equipment (e.g., portable diagnostic devices, utensils), and indirect costs (overhead). Medication costs were not included in the analysis as these vary significantly depending on the patients' diagnosis and condition.

#### **Step 4: Measurement of resource use for each resource group**

Resource use was systematically measured in relation to each type of treatment or care service delivered. For every relevant activity, we recorded the time spent by each category of healthcare professional, including time allocated to direct clinical care (such as TOBS and POCT), documentation, communication with collaborative partners, and transportation. Transportation time included travel to and from the patient’s home, as well as the transfer of blood samples and medications between the hospital and the home setting. Data were collected using micro-costing logs completed be healthcare staff, structured interviews, and, for data validation, we had data extracted from administrative records. Equipment utilisation was documented by interviewing staff in clinical departments, who provided information of device usage patterns and referred to local systems for verification.

#### **Step 5: Calculation of Capacity Cost Rate (CCR)**

*Staff costs:* For each staff group, annual salary expenditure was calculated and converted to an effective hourly rate according to Danish Medicines Council guidelines (1,122 effective hours/year). Overhead was added according to national and sector-specific standards: 18% for regional staff, 10% for municipal staff. The capacity cost rate (CCR) for each staff group was calculated by dividing the total annual staff cost (salary and overhead) by the practical capacity, defined as the number of hours realistically available for direct clinical work after adjustment for breaks, leave, and non-patient-facing duties.

*Equipment costs:* Equipment costs were determined by collecting comprehensive data on all relevant devices and materials used in HaH activities, including purchase price, depreciation, maintenance, and, where appropriate, consumables. Information on prices and acquisition costs was obtained directly from the departments of clinical engineering and from clinical personnel. Investment costs for equipment were converted to equivalent annual costs using standard annuitisation methods to account for the expected useful life of each item. The practical capacity of each device was defined as the expected number of operational hours per year, after allowing for required maintenance and repairs. The capacity cost rate for equipment was calculated by dividing the total annualised cost by the estimated available operational hours, consistent with international guidelines for economic evaluation in healthcare.

***Step 6: Analysing time measurements***

For each HaH activity, the principal time drivers were identified and systematically quantified. Detailed time estimates were primarily collected through micro-costing logs, which were maintained by municipal staff and recorded for each patient visit. These logs captured information on staff category, timing and duration of visits, the specific content of each activity, as well as time spent on communication, documentation, and transportation. To supplement the micro-costing logs and to provide comprehensive data on the use of equipment and specific activities, additional information was obtained through structured interviews with healthcare professionals.

#### **Step 7: Development of time equations**

#### In Step 7, activity-specific time equations were constructed to accurately represent the resource use associated with each component of HaH. For each relevant activity, such as direct patient care, communication, documentation, transport, and equipment use, we first identified the mean time spent per patient visit by each staff category, based on empirical observations and micro-costing logs. The proportion of visits or activities performed by each staff group was then established using the same dataset. These two elements, the mean time and staff proportion, were combined with the CCR for each staff group to generate an equation for each activity and staff type.

#### For example, the equation for direct patient care was defined as the meantime per visit spent by a given staff category, multiplied by the proportion of visits delivered by that group, and further multiplied by the relevant CCR. Similar equations were developed for communication, documentation, transport, and equipment, always using empirically observed time and staff distribution data together with the calculated CCRs. All time equations were validated by cross-checking against administrative records and staff interviews to ensure internal consistency and transparency.

#### The purpose of developing these activity- and staff-specific equations was to provide a reproducible and transparent basis for the subsequent calculation of total visit costs, allowing for detailed and accurate attribution of resource use.

#### **Step 8: Total cost calculation and tariff development**

In Step 8, the validated time equations and CCRs from the preceding step were systematically aggregated to estimate the total cost per HaH visit and to establish the structure for HaH tariffs. This calculation included all relevant resource components: direct patient care, communication (both initiated and received), documentation, and additional clinical activities, as well as equipment.

To accurately reflect variation in resource use and cost structure, all treatment tariff calculations were stratified by both time period and visit type. Specifically, tariffs were estimated separately for nine distinct time periods; weekday, Saturday, and Sunday/holiday, each subdivided into day, evening, and night, and for each time period, calculations were performed separately for first visits and following visits.

For each combination of time period and visit type, the treatment tariff was calculated as a weighted mean of costs for visits delivered by municipal home nurses and by acute nurses. The weights were determined by the observed proportion of visits performed by each staff type within the relevant subgroup, based on the micro-costing data.

For each staff type, the calculation incorporated the following activity components:

- time spent on the home visit
- time spent on communication (initiating and receiving)
- time spent on documentation

For each activity, the observed mean time per visit was multiplied by the relevant capacity cost rate (CCR) for staff salary, including overhead. These costs were summed across all activities and across staff types (using the appropriate weights for each subgroup).

In addition, the equipment cost per visit was calculated as the mean number of additional clinical activities performed per visit (within each subgroup), multiplied by a single, unified equipment CCR (estimated across all staff groups and time periods).

The resulting treatment tariff for each combination of time period and visit type was therefore calculated as follows:

$$Tariff_{\left( treatment,p,v \right)}=\sum_{n} w_{\left( n,p,v \right)}\left[ \sum_{r} \left( T_{\left( visit,r,p,v,n \right)}+T_{\left( comm,init,r,p,v,n \right)}+T_{\left( comm,rec,r,p,v,n \right)}+T_{\left( doc,r,p,v,n \right)} \right)\times CCR_{\left( salary,r \right)} \right]+\sum_{a} \left( N_{\left( a,p,v \right)}\times CCR_{a} \right)$$

Where:

- *p* = time period (one of nine categories: weekday, Saturday, or Sunday/holiday; each subdivided into day, evening, or night)
- *v* = visit type (first visit or following visit)
- *n* = visit provider type (acute-led or home-led visit); for first visits, *n* = acute
- *w₍*_n,p,v_*₎* = observed proportion of visits delivered by provider type n within time period p and visit type v; for first visits, *w*₍_acute,p,first_₎ = 1
- *r* = staff category involved in the visit episode (e.g., acute nurse, home nurse, hospital nurse, ED/MD physician)
- *T*₍_visit,r,p,v,n_₎ = mean time spent by staff category *r* on direct patient treatment and preparation for visits of type *v* delivered by provider type *n* in time period *p*
- *T*₍_comm,init,r,p,v,n_₎ = mean time spent by staff category *r* initiating communication (e.g., contacting other healthcare professionals)
- *T*₍_comm,rec,r,p,v,n_₎ = mean time spent by staff category *r* receiving and responding to communication
- *T*₍_doc,r,p,v,n_₎ = mean time spent by staff category *r* on documentation after the visit
- *CCR*₍_salary,r_₎ = capacity cost rate for staff category *r*, including salary and overhead
- *a* = clinical activity (e.g., POCT-CRP, TOBS, venflon, IV treatment)
- *N*₍_a,p,v_₎ = mean frequency of activity *a* per visit within time period *p* and visit type *v*
- *CCR*_a_= capacity cost rate (unit cost) associated with activity *a*, including equipment and consumables as applicable

The transport component was estimated using time data for both municipal home nurses and acute nurses, with staff transport time multiplied by the relevant wage rate. These were then combined as a weighted mean, reflecting the relative proportion of transports undertaken by each staff type during each time period. To estimate the travel cost per visit, observed driving times were converted to distance using an assumed average speed of 40 km/h, and the resulting distance was multiplied by the low government-set mileage reimbursement rate. This travel cost was then added to the staff transportation cost to form the total transport component for each visit.

$$C_{\left( transport,p \right)}=\sum_{n} w_{\left( n,p \right)}\left( T_{\left( trans,n,p \right)}\times CCR_{\left( salary,n \right)} \right)+\left( D_{\left( mean,p \right)}\times R_{\left( travel \right)} \right)$$

Where:

- *p* = time period (one of nine categories: weekday, Saturday or Sunday/holiday × day, evening or night)
- *n* = visit provider type (acute-led or home-led visit)
- w_(n,p)_ = observed proportion of transport performed by provider type n within time period p
- T₍_trans,n,p_₎ = mean transport time per visit for provider type n in time period p
- CCR₍_salary,n_₎ = capacity cost rate for staff salary (including overhead) for provider type n
- D₍_mean,p_₎ = estimated mean driving distance per visit in time period p, calculated from observed transport time and an assumed average speed of 40 km/h
- R₍_travel_₎ = government-set mileage reimbursement rate (low tariff)

Transportation costs were calculated for each of the three municipalities to explore potential variation due to geography or organisational structure.

Finally, the treatment tariff estimates for each combination of time period and visit type (first visit and following visit) were aggregated to generate a single overall tariff for first visits and a single overall tariff for following visits. This aggregation was performed by calculating a weighted mean across all time periods, using the observed distribution of first and following visits in the study population.

The transport component was similarly summarised as a single aggregate tariff, calculated as a weighted mean across all time periods and municipalities, based on the empirical distribution of visits and transport activities.

The final tariffs for first visits and following visits were calculated as the sum of the respective aggregated treatment tariff and the transport component. In this way, the analysis resulted in two final tariffs; one for first visits and one for following visits, each reflecting the total expected cost per visit, including both treatment and transport.
