# Supplementary material for: Developing Hospital at Home tariffs in Denmark: a time-driven activity-based microcosting approach within a randomised controlled trial
Source: BMJ Open. 2026 Apr 20;16(4):e113738. doi: 10.1136/bmjopen-2025-113738 (PMC13110545; doi:10.1136/bmjopen-2025-113738)
Supplement: online supplemental file 2 [file bmjopen-16-4-s002.docx]

Supplementary file 2. Unit costs staff (€2025)

Hourly capacity cost rates (including employer contributions and overhead at 10% for municipal staff and 18% for regional staff in the base case).

Nurses

|  | Monthly salary | Hourly salary including salary supplements | | | | | | | | |
| --- | --- | --- | --- | --- | --- | --- | --- | --- | --- | --- |
|  |  | *Weekday* | | | *Saturday* | | | *Sunday/holiday* | | |
| Resources |  | *Day* | *Evening* | *Night* | *Day* | *Evening* | *Night* | *Day* | *Evening* | *Night* |
| Acute nurse (municipality) | 6,716 | 71 | 93 | 96 | 93 | 114 | 118 | 107 | 129 | 132 |
| Home nurse (municipality) | 6,421 | 68 | 89 | 92 | 89 | 109 | 113 | 102 | 123 | 126 |
| Hospital nurse | 6,533 | 69 | 92 | 96 | 92 | 114 | 118 | 102 | 124 | 128 |

Hospital physician

| Resource | Monthly salary | Effective hourly salary |
| --- | --- | --- |
| ED/MD physician | 14,884 | 158 |

Unit cost – general practitioner

|  | *Weekday* | |  | *Saturday* | |  | *Sunday/holiday* | | |
| --- | --- | --- | --- | --- | --- | --- | --- | --- | --- |
| Resource | Day | *Evening* | *Night* | *Day* | *Evening* | *Night* | *Day* | *Evening* | *Night* |
| General practitioner (telephone consultation tariff) | 7.45 | 10.39 | 17.20 | 10.39 | 13.56 | 17.20 | 13.56 | 13.56 | 17.20 |
