# Supplementary material for: Developing Hospital at Home tariffs in Denmark: a time-driven activity-based microcosting approach within a randomised controlled trial
Source: BMJ Open. 2026 Apr 20;16(4):e113738. doi: 10.1136/bmjopen-2025-113738 (PMC13110545; doi:10.1136/bmjopen-2025-113738)
Supplement: online supplemental file 3 [file bmjopen-16-4-s003.docx]

Supplementary File 3. Calculation of Capacity Cost Rate of equipment

| Test/equipment | Investment | Lifetime equipment (Years) | Annual cost (equipment) | Annual use (capacity) | Capacity cost rate per unit (CCR) | Total Capacity Cost Rate (CCR) |
| --- | --- | --- | --- | --- | --- | --- |
| **POCT-CRP** |  |  |  |  |  |  |
| Equipment | 715 | 9 | 94 | 100 | 0.94 |  |
| 50 pre-filled cuvettes for CRP and Hgb measurement | 127 |  |  |  | 2.53 | 3.47 |
| **TOBS** |  |  |  |  |  |  |
| Blood pressure monitor | 53 | 9 | 7 | 300 | 0.02 |  |
| Pulse oximeter | 29 | 2 | 15 | 300 | 0.05 |  |
| Thermometer (ear) | 65 | 2 | 34 | 300 | 0.11 | 0.19 |
| **Leukocyte analyser** | 988 | 5 | 219 | 25 | 8.75 |  |
| Cuvettes (25 pcs) | 84 |  |  |  | 3.37 | 12.12 |
| **Haemoglobin analyser** | 772 | 5 | 171 | 50 | 3.42 |  |
| Hb Cuvettes (25 pcs) | 22 |  |  |  | 0.89 | 4.31 |
| **ECG** |  |  |  |  |  |  |
| ECG equipment | 3380 | 9 | 444 | 10 | 44.43 |  |
| ECG electrodes (300 pcs) | 59 |  |  |  | 1.98 | 46.41 |
| **Blood glucose meter** | 13 | 2 | 7 | 50 | 0.14 |  |
| Test strips (50 pcs) | 6 |  |  |  | 0.12 | 0.25 |
| **Urine analysis equipment** | 652 | 5 | 144 | 100 | 1.44 |  |
| Test strips (100 pcs) | 14 |  |  |  | 0.14 | 1.59 |
| **Bladder scanner** | 5200 | 9 | 684 | 5 | 136.70 | 136.70 |
| **Blood sample** |  |  |  |  |  |  |
| Transportation kit | 73 | 4 |  | 200 | 0.64 |  |
| Bag | 52 | 2 | 27 | 200 | 0.14 |  |
| Analysis (at hospital including utencils) |  |  |  |  | 14.64 | 15.42 |
| **CAD** |  |  |  |  |  |  |
| Catheters (10 pcs) | 28 |  |  |  | 2.76 | 2.76 |
| **Venflon** |  |  |  |  |  |  |
| IV cannula blue (100 pcs) | 68 |  |  |  | 0.68 |  |
| Infusion sets (100 pcs) | 188 |  |  |  | 1.88 |  |
| Saline syringes (90 pcs) | 34 |  |  |  | 0.34 |  |
| Codan extension tubing with membrane (100 pcs) | 254 |  |  |  | 2.82 |  |
| Injection wipes (100 pcs) | 1 |  |  |  | 0.05 |  |
| Fixation tape for venflon (100 pcs) | 13 |  |  |  | 0.27 |  |
| Fixation bandages for venflon (20 pcs) | 1 |  |  |  | 0.09 |  |
| Absorbent bandages (100 pcs) | 3 |  |  |  | 0.05 | 6.18 |
| **IV treatment** |  |  |  |  |  |  |
| Infusion sets (100 pcs) (used every 2'nd treatment) | 188 |  |  |  | 0.9 |  |
| Natriumchlorid syringe (30 pcs) | 70 |  |  |  | 2.3 | 3.3 |

*CCR, Capacity Cost Rate, representing the cost per unit of time or use for a given resource (e.g. equipment or staff); POCT-CRP, Point-of-Care Test for C-Reactive Protein, a rapid bedside blood test used to assess inflammation; Hgb, Haemoglobin, a protein in red blood cells that carries oxygen; TOBS, Triage and Observation of Vital Signs, including pulse, consciousness, temperature, respiration, and systolic blood pressure; ECG, Electrocardiogram, a diagnostic test that records the electrical activity of the heart; CRP, C-Reactive Protein, an inflammatory marker measured to assess infection or inflammation; IV, Intravenous, referring to the administration of fluids or medications directly into a vein; CAD, Catheter-Associated Device, used here for urinary or venous catheterisation equipment.*
