# Supplementary material for: Developing Hospital at Home tariffs in Denmark: a time-driven activity-based microcosting approach within a randomised controlled trial
Source: BMJ Open. 2026 Apr 20;16(4):e113738. doi: 10.1136/bmjopen-2025-113738 (PMC13110545; doi:10.1136/bmjopen-2025-113738)
Supplement: online supplemental file 4 [file bmjopen-16-4-s004.docx]

Supplementary file 4. Ressource inputs

|  |  |  |  |  |  |  |  |  |  |  |
| --- | --- | --- | --- | --- | --- | --- | --- | --- | --- | --- |
|  |  | Time per visit (hours) | | | | | | | | |
| **FIRST VISIT** |  | *Weekday* | | | *Saturday* | | | *Sunday/holiday* | | |
|  |  | *Day* | *Evening* | *Night* | *Day* | *Evening* | *Night* | *Day* | *Evening* | *Night* |
| Acute nurse visit | Acute nurse | 2.518 | 2.078 | 0.000 | 1.433 | 1.920 | 3.225 | 2.415 | 2.147 | 0.000 |
|  | Home nurse | 0.005 | 0.000 | 0.000 | 0.000 | 0.000 | 0.000 | 0.000 | 0.000 | 0.000 |
|  | Hospital nurse | 0.025 | 0.005 | 0.000 | 0.000 | 0.085 | 0.000 | 0.000 | 0.000 | 0.000 |
|  | ED/MD physician | 0.151 | 0.117 | 0.000 | 0.096 | 0.210 | 0.125 | 0.250 | 0.142 | 0.000 |
|  |  |  |  |  |  |  |  |  |  |  |
|  |  |  |  |  |  |  |  |  |  |  |
|  |  | Time per visit (hours) | | | | | | | | |
| **FOLLOWING VISITS** | | *Weekday* | | | *Saturday* | | | *Sunday/holiday* | | |
|  |  | *Day* | *Evening* | *Night* | *Day* | *Evening* | *Night* | *Day* | *Evening* | *Night* |
| Acute nurse visit | Acute nurse | 1.147 | 0.835 | 0.881 | 1.045 | 0.906 | 1.083 | 0.995 | 0.791 | 1.035 |
|  | Home nurse | 0.025 | 0.002 | 0.002 | 0.001 | 0.002 | 0.000 | 0.008 | 0.003 | 0.000 |
|  | Hospital nurse | 0.003 | 0.001 | 0.000 | 0.000 | 0.011 | 0.000 | 0.001 | 0.000 | 0.007 |
|  | ED/MD physician | 0.125 | 0.026 | 0.006 | 0.109 | 0.032 | 0.009 | 0.087 | 0.029 | 0.007 |
| Home nurse visit | Acute nurse | 0.010 | 0.011 | 0.000 | 0.000 | 0.000 | 0.000 | 0.009 | 0.000 | 0.006 |
|  | Home nurse | 0.867 | 0.854 | 0.757 | 0.852 | 0.818 | 0.773 | 0.787 | 0.988 | 0.763 |
|  | Hospital nurse | 0.005 | 0.002 | 0.000 | 0.012 | 0.000 | 0.000 | 0.000 | 0.017 | 0.000 |
|  | ED/MD physician | 0.001 | 0.000 | 0.000 | 0.000 | 0.000 | 0.000 | 0.000 | 0.011 | 0.000 |
|  |  |  |  |  |  |  |  |  |  |  |
|  |  |  |  |  |  |  |  |  |  |  |
|  |  | Activities | | | | | | | | |
| **FIRST VISIT** |  | *Weekday* | | | *Saturday* | | | *Sunday/holiday* | | |
|  |  | *Day* | *Evening* | *Night* | *Day* | *Evening* | *Night* | *Day* | *Evening* | *Night* |
|  | POCT-CRP | 0.371 | 0.170 | 0.000 | 0.000 | 0.000 | 0.500 | 0.000 | 0.167 | 0.000 |
|  | TOBS | 0.771 | 0.698 | 0.000 | 0.571 | 1.000 | 1.000 | 0.500 | 0.667 | 0.000 |
|  | Leukocyte analysis | 0.000 | 0.000 | 0.000 | 0.000 | 0.000 | 0.500 | 0.000 | 0.000 | 0.000 |
|  | ECG | 0.000 | 0.000 | 0.000 | 0.000 | 0.000 | 0.000 | 0.000 | 0.000 | 0.000 |
|  | Blood glucose analysis | 0.000 | 0.000 | 0.000 | 0.000 | 0.000 | 0.000 | 0.000 | 0.000 | 0.000 |
|  | Urine analysis | 0.114 | 0.094 | 0.000 | 0.000 | 0.000 | 0.000 | 0.000 | 0.167 | 0.000 |
|  | Bladder scan | 0.000 | 0.000 | 0.000 | 0.000 | 0.000 | 0.000 | 0.000 | 0.000 | 0.000 |
|  | Blood sample | 0.714 | 0.585 | 0.000 | 0.286 | 0.500 | 1.000 | 0.500 | 0.833 | 0.000 |
|  | CAD | 0.000 | 0.000 | 0.000 | 0.000 | 0.000 | 0.000 | 0.000 | 0.000 | 0.000 |
|  | Venflon | 0.771 | 0.698 | 0.000 | 0.286 | 0.500 | 1.000 | 0.500 | 0.667 | 0.000 |
|  | IV-treatment | 0.629 | 0.925 | 0.000 | 0.714 | 0.500 | 1.000 | 1.000 | 0.833 | 0.000 |
|  | GP telephone consultation | 0.057 | 0.000 | 0.000 | 0.000 | 0.000 | 0.000 | 0.000 | 0.000 | 0.000 |
|  |  |  |  |  |  |  |  |  |  |  |
|  |  |  |  |  |  |  |  |  |  |  |
|  |  | Activities | | | | | | | | |
| **FOLLOWING VISITS** | | *Weekday* | | | *Saturday* | | | *Sunday/holiday* | | |
|  |  | *Day* | *Evening* | *Night* | *Day* | *Evening* | *Night* | *Day* | *Evening* | *Night* |
|  | POCT-CRP | 0.383 | 0.028 | 0.014 | 0.295 | 0.023 | 0.083 | 0.314 | 0.022 | 0.067 |
|  | TOBS | 0.459 | 0.173 | 0.139 | 0.397 | 0.227 | 0.167 | 0.430 | 0.089 | 0.067 |
|  | Leukocyte analysis | 0.034 | 0.000 | 0.014 | 0.026 | 0.000 | 0.000 | 0.070 | 0.022 | 0.000 |
|  | ECG | 0.002 | 0.000 | 0.000 | 0.000 | 0.000 | 0.000 | 0.000 | 0.000 | 0.000 |
|  | Blood glucose analysis | 0.002 | 0.000 | 0.000 | 0.000 | 0.000 | 0.000 | 0.000 | 0.000 | 0.000 |
|  | Urine analysis | 0.007 | 0.004 | 0.000 | 0.013 | 0.000 | 0.000 | 0.012 | 0.000 | 0.067 |
|  | Bladder scan | 0.010 | 0.000 | 0.000 | 0.000 | 0.000 | 0.000 | 0.000 | 0.000 | 0.067 |
|  | Blood sample | 0.214 | 0.063 | 0.014 | 0.231 | 0.045 | 0.083 | 0.140 | 0.000 | 0.067 |
|  | CAD | 0.002 | 0.000 | 0.000 | 0.000 | 0.000 | 0.000 | 0.000 | 0.000 | 0.000 |
|  | Venflon | 0.107 | 0.157 | 0.097 | 0.103 | 0.091 | 0.000 | 0.081 | 0.089 | 0.133 |
|  | IV-treatment | 0.684 | 0.839 | 0.986 | 0.731 | 0.909 | 1.000 | 0.767 | 1.000 | 1.067 |
|  | GP telephone consultation | 0.024 | 0.004 | 0.014 | 0.000 | 0.000 | 0.000 | 0.000 | 0.000 | 0.000 |

|  |  | Number of visits | | | | | | | | | |
| --- | --- | --- | --- | --- | --- | --- | --- | --- | --- | --- | --- |
|  |  | *Weekday* | | | *Saturday* | | | *Sunday/holiday* | | | Totals |
|  |  | *Day* | *Evening* | *Night* | *Day* | *Evening* | *Night* | *Day* | *Evening* | *Night* |  |
| **First visits** | Acute nurse | 35 | 53 | 0 | 7 | 2 | 2 | 2 | 6 | 0 | 107 |
| **Following visits** | Acute nurse | 345 | 209 | 62 | 64 | 33 | 9 | 71 | 30 | 12 | 835 |
|  | Home nurse | 67 | 45 | 10 | 14 | 11 | 3 | 15 | 15 | 3 | 183 |
|  | Total (following visits) | 412 | 254 | 72 | 78 | 44 | 12 | 86 | 45 | 15 | 1018 |
