# Supplementary material for: Developing Hospital at Home tariffs in Denmark: a time-driven activity-based microcosting approach within a randomised controlled trial
Source: BMJ Open. 2026 Apr 20;16(4):e113738. doi: 10.1136/bmjopen-2025-113738 (PMC13110545; doi:10.1136/bmjopen-2025-113738)
Supplement: online supplemental file 5 [file bmjopen-16-4-s005.docx]

Supplementary file 5. Example calculation of tariff (following visit, weekday day)

| **Weekday day** |  |  |  |  |  |  |
| --- | --- | --- | --- | --- | --- | --- |
|  | **Visits (n: 412)** | **Weight** | **Staff category** | **Total time per visit (hours)** | **Salary (per hour)** | **Staff cost per visit** |
| Acute nurse visit |  |  | Acute nurse | 1.15 | 71.45 | 81.98 |
|  |  |  | Home nurse | 0.03 | 68.30 | 1.72 |
|  |  |  | Hospital nurse | 0.00 | 69.50 | 0.18 |
|  |  |  | ED/MD physician | 0.12 | 158.34 | 19.73 |
| Total staff cost (acute nurse visit) | 345 | 0.837 |  |  |  | 103.60 |
|  |  |  |  |  |  |  |
| Home nurse visit |  |  | Acute nurse | 0.01 | 71.45 | 0.71 |
|  |  |  | Home nurse | 0.87 | 68.30 | 59.21 |
|  |  |  | Hospital nurse | 0.00 | 69.50 | 0.34 |
|  |  |  | ED/MD physician | 0.00 | 158.34 | 0.19 |
| Total staff cost (home nurse visit) | 67 | 0.163 |  |  |  | 60.46 |
|  |  |  |  |  |  |  |
| Weighted total staff cost per visit |  |  |  |  |  | 96.59 |
|  |  |  | **Treatment activity** | **Number per visit** | **Capacity cost rate** | **Activity cost per visit** |
|  |  |  | POCT-CRP | 0.383 | 3.47 | 1.33 |
|  |  |  | TOBS | 0.459 | 0.19 | 0.09 |
|  |  |  | Leukocyte analysis | 0.034 | 12.12 | 0.41 |
|  |  |  | ECG | 0.002 | 46.41 | 0.11 |
|  |  |  | Blood glucose analysis | 0.002 | 0.25 | 0.00 |
|  |  |  | Urine analysis | 0.007 | 1.59 | 0.01 |
|  |  |  | Bladder scan | 0.010 | 136.70 | 1.33 |
|  |  |  | Blood sample | 0.214 | 15.42 | 3.29 |
|  |  |  | CAD | 0.002 | 2.76 | 0.01 |
|  |  |  | Venflon | 0.107 | 6.18 | 0.66 |
|  |  |  | IV-treatment | 0.684 | 3.28 | 2.24 |
|  |  |  | GP telephone consultation | 0.024 | 7.45 | 0.18 |
| Total activity cost per visit |  |  |  |  |  | 9.67 |
| Total cost per visit |  |  |  |  |  | **106.26** |

This value (106.26) represents the unweighted treatment cost for follow-up visits conducted during weekday daytime only. The overall mean treatment cost reported in Table 2 reflects a weighted average across all nine time periods.
