# Supplementary material for: Developing Hospital at Home tariffs in Denmark: a time-driven activity-based microcosting approach within a randomised controlled trial
Source: BMJ Open. 2026 Apr 20;16(4):e113738. doi: 10.1136/bmjopen-2025-113738 (PMC13110545; doi:10.1136/bmjopen-2025-113738)
Supplement: online supplemental file 6 [file bmjopen-16-4-s006.docx]

Supplementary file 6. Calculation of the transportation component

|  | **Skive** | | | | | | **Silkeborg** | | | | | |
| --- | --- | --- | --- | --- | --- | --- | --- | --- | --- | --- | --- | --- |
|  | **n** | **Weight** | **Mean time (min)** | **Time component** | **Staff transportation cost** | **Cost component** | **n** | **Weight** | **Mean time (min)** | **Time component** | **Staff transportation cost** | **Cost component** |
|  |  |  | *Per visit* | *Weighted* | *Per visit* | *Weighted* |  |  | *Per visit* | *Weighted* | *Per visit* | *Weighted* |
| **Weekday** |  |  |  |  |  |  |  |  |  |  |  |  |
| *day* | 31 | 0.27 | 107 | 29 | 100.41 | 27.07 | 39 | 0.25 | 151 | 38 | 109.23 | 27.48 |
| *evening* | 27 | 0.23 | 86 | 20 | 88.14 | 20.69 | 32 | 0.21 | 109 | 22 | 108.69 | 22.44 |
| *night* | 4 | 0.03 | 76 | 3 | 89.92 | 3.13 | 19 | 0.12 | 53 | 6 | 89.22 | 10.94 |
| **Saturday** |  |  |  |  |  |  |  |  |  |  |  |  |
| *day* | 14 | 0.12 | 37 | 4 | 43.61 | 5.31 | 17 | 0.11 | 44 | 5 | 60.62 | 6.65 |
| *evening* | 10 | 0.09 | 34 | 3 | 68.54 | 5.96 | 8 | 0.05 | 45 | 2 | 66.51 | 3.43 |
| *night* | 3 | 0.03 | 22 | 1 | 41.32 | 1.08 | 7 | 0.05 | 35 | 2 | 68.50 | 3.09 |
| **Sunday** |  |  |  |  |  |  |  |  |  |  |  |  |
| *day* | 16 | 0.14 | 38 | 5 | 50.91 | 7.08 | 16 | 0.10 | 51 | 5 | 70.21 | 7.25 |
| *evening* | 8 | 0.07 | 32 | 2 | 90.87 | 6.32 | 11 | 0.07 | 43 | 3 | 92.75 | 6.58 |
| *night* | 2 | 0.02 | 23 | 0 | 48.44 | 0.84 | 6 | 0.04 | 31 | 1 | 61.57 | 2.38 |
|  |  |  |  |  |  |  |  |  |  |  |  |  |
| **Total** | 115 | 1 |  | 68 |  | **77.48** | 155 | 1 |  | 85 |  | **90.25** |
| **Total across sites (n 495)** |  | 0.23 |  | 16 |  | 18.00 |  | 0.31 |  | 27 |  | 28.26 |
|  |  |  |  |  |  |  |  |  |  |  |  |  |
|  | **Viborg** | | | | | |  | **All municipalities** | | | | |
|  | **n** | **Weight** | **Mean time (min)** | **Time component** | **Staff transportation cost** | **Cost component** |  | **Mean transportation time (min)** | **Mean staff transportation cost** | **Mean travel distance (km)** | **Mean transportation cost** | **Mean total transportation cost** |
|  |  |  | *Per visit* | *Weighted* | *Per visit* | *Weighted* |  | *Weighted* | *Weighted* | *40 km per hour* | *Low rate** |  |
| **Weekday** |  |  |  |  |  |  |  |  |  |  |  |  |
| *day* | 61 | 0.27 | 105 | 29 | 95.55 | 25.91 |  |  |  |  |  |  |
| *evening* | 56 | 0.25 | 95 | 24 | 124.58 | 31.01 |  |  |  |  |  |  |
| *night* | 17 | 0.08 | 47 | 4 | 76.12 | 5.75 |  |  |  |  |  |  |
| **Saturday** |  |  |  |  |  |  |  |  |  |  |  |  |
| *day* | 28 | 0.12 | 43 | 5 | 54.10 | 6.73 |  |  |  |  |  |  |
| *evening* | 16 | 0.07 | 46 | 3 | 86.88 | 6.18 |  |  |  |  |  |  |
| *night* | 3 | 0.01 | 60 | 1 | 117.24 | 1.56 |  |  |  |  |  |  |
| **Sunday** |  |  |  |  |  |  |  |  |  |  |  |  |
| *day* | 23 | 0.10 | 46 | 5 | 69.57 | 7.11 |  |  |  |  |  |  |
| *evening* | 16 | 0.07 | 61 | 4 | 125.85 | 8.95 |  |  |  |  |  |  |
| *night* | 5 | 0.02 | 40 | 1 | 88.56 | 1.97 |  |  |  |  |  |  |
|  |  |  |  |  |  |  |  |  |  |  |  |  |
| **Total** | 225 | 1 |  | 75 |  | **95.17** |  |  |  |  |  |  |
| **Total across sites (n 495)** |  | 0.45 |  | 34 |  | 43.26 |  | **77** | **89.52** | **51** | **14.81** | **104.32** |
