# Supplementary material for: Developing Hospital at Home tariffs in Denmark: a time-driven activity-based microcosting approach within a randomised controlled trial
Source: BMJ Open. 2026 Apr 20;16(4):e113738. doi: 10.1136/bmjopen-2025-113738 (PMC13110545; doi:10.1136/bmjopen-2025-113738)
Supplement: Supplementary file 7 [file bmjopen-2025-113738.reviewer_comments.pdf]

PEER REVIEW HISTORY

BMJ Open publishes all reviews undertaken for accepted manuscripts. Reviewers are asked to complete a checklist review form and are provided with free text boxes to elaborate on their assessment. These free text comments are reproduced below.

ARTICLE DETAILS

Title (Provisional)

Developing Hospital at Home Tariffs in Denmark: A Time-Driven Activity-Based Micro-Costing Approach within a Randomised Controlled Trial

Authors

Risør, Bettina Wulff; Duvald, Iben; Nielsen, Camilla Palmhøj; Tayyari, Nasrin

---

VERSION 1 - REVIEW

**Reviewer**	**1**
**Name**	**Dack, Kyle**
**Affiliation**	**University of Bristol, MRC Integrative Epidemiology Unit**
**Date**	**25-Dec-2025**
**COI**	**None**

Statistical review summary:

This is an interesting descriptive micro-costing analysis, which produced valuable findings. The design and statistical analysis follow best practices and I have no concerns or suggestions – it is a very well-conducted study. There is no relevant reporting guideline for this type of study (CHEER is the closest, but approximately half the items would not be relevant), so I have marked that item as NA. But in my opinion the methods and reporting are clear and transparent, with no major omissions.

Abstract:

1. The abstract gives a clear and comprehensive overview of the study. My only suggestion is to add the study dates, so that abstract readers can see this is an up-to-date analysis.

Introduction:

2. The introduction is clear, and gives a good overview of microcosting for those who may be unfamiliar with it.

Methods:

3. Study design – it may be helpful to state the study dates which this analysis is based upon in the first paragraph. This information is provided, but quite a long way into the methods.

4. Participants – It sounds like there were no further exclusion criteria, other than those already applied in the RCT. But I suggest directly confirming this in the participants section, to avoid uncertainty.

5. “A treatment episode was defined as the full HaH course from admission to discharge” what is meant by discharge in this context, given that patients remained at home?

6. The description of the RCT, the participants, and data source is all very clear. It would be helpful if the reference to the micro-costing development paper was available (bmjopen-2025-106799). Hopefully that paper will be published in-time to be referenced but this may be beyond the authors control.

7. Was patient-level clustering taken into account – the variation in the number of visits per patient? For example, identifying the mean per patient, and then using these means to calculate the overall mean. I do not think this is essential, but it could be helpful if there is a small group of patients with a very high number of visits (because raw mean would over-represent these patients).

Results

8. Related to the above comment, I suggest adding more details about the distribution of visits, given that this is the core unit of analysis. The mean and SD is reported, but was this normally distributed? The median, potentially min/max, could be reported in-text. This would help readers to interpret the findings.

9. Generally the results are very clear. I checked tables, statistics, and found no inconsistencies or ambiguities. The figures are also well-presented.

Discussion:

10. The authors discuss the omission of medication costs in the limitations section. I agree that this decision likely strengthens the reliability of the findings, given the high heterogeneity in this type of cost. However, for readers unfamiliar with this type of study, it may be helpful to also state that this is a common exclusion in micro-costing studies.

11. The limitation statement regarding repeated measures answers my earlier query regarding whether these were taken into account in the calculations. The justification is reasonable, but adding more details of the distribution of visits would help readers to understand the impact of this. The mean and SD of visits is provided in Table 1, but adding the median and min/max range (or even a histogram in a supplement) would help to understand if outliers with extremely high visit counts had much impact or not.

Reviewer 2

Name Bjørnelv, Gudrun

Affiliation Norwegian University of Science and Technology, Department of public health and nursing

**Date**	**17-Feb-2026**
**COI**	**None**

Thank you for giving me the opportunity to read this study where the authors have used a time-driven activity-based micro-costing approach to estimate a hospital-at-home tariff in Denmark. Overall, this is a well-conducted and interesting study. Getting better estimates of the costs in the home- and community based care setting is very important now and in the future when municipalities will bear more of the burden of the aging population. High-quality cost-effectiveness studies will then be warranted, and in these, having good methods to estimate costs also in other settings that in the hospital is important. This study is a timely and warranted contribution to enable this.

I primarily have minor comments that I think would increase the value of the study. Generally, the manuscript is well written and methods seem sound. I think you could get a bit more out of the study by i.e., making a prediction model based on the numbers in the study. This could be a flexible tool to estimate costs of different types of visits (depending on which input variables you 'turn up or down' (see comment below). The papers weakest part is the discussion section, where I think the authors can go more in-depth of their own methods, analyses and findings compared to the broader literature to better contextualize the findings.

My feedback is provided in a point-by-point list below.

1. TDABC is several places written out as Trime-Driven Activity-Based Costing with the abbreviation in parentheses – please write the full definition only the first time it is mentioned and use the abbreviation thereafter.

2. To be able to have a hospital-at-home, I suppose there is a need for a certain capacity that can react if acute episodes occur. This means that persons will have to be on call, but without actually providing care. It was not clear how you distributed this time to the treatments?

3. For the point estimates of the tariff (i.e., the cost per fist (€237.67) and following (€98,30) visit) – why is there no uncertainty in the estimates? I would have expected to see for example 95% confidence intervals surrounding the point estimates? This is the case for all point estimates. If I understand the methods correct, you get these point-estimates based on the equations (1 and 2 depending on whether it is the visits or transport costs you estimate). Using bootstrapping would enable you to make estimates of uncertainties (confidence bounds).

4. In the text (result section), although you have a lot of the numbers in the tables, I think the read would be better if you also have some numbers in the text. For example, "First visits most frequently occurred on weekday evenings (50%) and days (33%)…etc).

5. It would be interesting to see how different patients conditions influence the cost-estimates (i.e., are the costs different depending on whether the patient has pneumonia, acute cellulitis, UTI or other diagnoses?)

6. For table 2, I think it would be interesting with more information on the different cost components that make up the finale estimates. Could you make a table where you show the average resource use (i.e., time spent for different professions) and unit costs? Then the reader/ user can estimate the total costs themselves by timing the average with the unit cost. This would make the work much more transparent! Now there is a 'black box' since you do not indicate actual average resource use and unit costs that go into the equations 1 and 2.

7. Also, aligned with the question above – it should be possible for the reader to understand the numbers going into the equations 1 and 2. So: could you make a prediction model where readers/ users of the work can vary different factors in the equation up and down, and see how this influences the findings? Make a flexible prediction model of the cost per visit? For example, what happens if the time spent by nurses increases or decreases? Or if their salary increases/ decreases? This would also enable people to adjust both the unit and the unit costs according to their setting (i.e., if using your model in another country such as Sweden or Norway).

8. In Table 4, I do not understand the headings (e.g., why does it for the overhead costs say 0%/0%, 10%/18% and 15%/25%?). I understand it is low, base case and high, but why are there two numbers for low/ high?

9. When you show the application of the tariff – can you include the actual calculations (so how do you come to €2,386.67, €2,590.97 and € 4,225.37? And, are the decimal numbers necessary?

10. In the discussion you start by saying that you developed and validated this – I see you have developed it using sound methods. But how was it validated?

11. Overall, the discussion section is weak. The first two pages are completely without any references or reflections related to other literature – and the rest is too weak. I think you should exploit the discussion section to really dig deeper into the comparison to other methods, other findings (what are the estimated costs for HAH in the literature) ++ In hospitals, for example, we use diagnosis-related groups and have methods to estimate costs per DRG. How are the methods used there different/ similar to your methods? Often, cost-effectiveness analyses includes treatment in hospital and in the municipal setting – if methods used to estimate costs in the hospital differs greatly from those used to estimate costs in the municipality this might be problematic. This should be elaborated.

12. Also, I would like to have a broader discussion on the applicability of the methods to other municipal interventions – how should this be applied, updated and rolled out to enable municipalities to have better control over the costs of the care they provide?

13. More elaborate discussion (except for what I suggest) is also warranted.

14. Also, I do not see any information of the ethics in the study, this should be included before the result section.

VERSION 1 - AUTHOR RESPONSE

Reviewer: 1
Dr. Kyle Dack, University of Bristol
Comments to the Author:
Statistical review summary:
This is an interesting descriptive micro-costing analysis, which produced valuable findings. The design and statistical analysis follow best practices and I have no concerns or suggestions – it is a very well-conducted study. There is no relevant reporting guideline for this type of study (CHEER is the closest, but approximately half the items would not be relevant), so I have marked that item as NA. But in my opinion the methods and reporting are clear and transparent, with no major omissions.

Comment	*Response*
Abstract:<br>1. The abstract gives a clear and comprehensive overview of the study. My only suggestion is to add the study dates, so that abstract readers can see this is an up-to-date analysis.	We thank the reviewer for this suggestion. The study period has now been added to the abstract to clarify the timeframe of data collection and to emphasise that the analysis is based on recent empirical data.
Introduction:<br>2. The introduction is clear, and gives a good overview of microcosting for those who may be unfamiliar with it.	We thank the reviewer for this positive assessment of the Introduction.
Methods:<br>3. Study design – it may be helpful to state the study dates which this analysis is based upon in the first paragraph. This information is provided, but quite a long way into the methods.	We thank the reviewer for this suggestion. The study period for the micro-costing analysis has now been specified in the first paragraph of the Methods section to improve clarity and transparency (p. 5).
4. Participants – It sounds like there were no further exclusion criteria, other than those already applied in the RCT. But I suggest directly confirming this in the participants section, to avoid uncertainty.	We thank the reviewer for this suggestion. We have now explicitly clarified in the Participants section that no additional inclusion or exclusion criteria were applied beyond those defined in the RCT protocol (p. 6):

	"No additional inclusion or exclusion criteria were applied for the micro-costing study beyond those defined in the RCT protocol."
5. "A treatment episode was defined as the full HaH course from admission to discharge" what is meant by discharge in this context, given that patients remained at home?	We thank the reviewer for highlighting this point. We have clarified that "discharge" refers to the formal termination of the HaH treatment episode, after which responsibility for care reverted to usual primary or municipal services. (p. 6)<br><br>*"A treatment episode was defined as the full HaH course from admission to formal termination of the HaH treatment, after which responsibility reverted to usual primary or municipal care services. Episodes were also considered terminated if the patient required escalation of care and was converted to conventional hospital admission, at which point the HaH episode ended."*
6. The description of the RCT, the participants, and data source is all very clear. It would be helpful if the reference to the micro-costing development paper was available (bmjopen-2025-106799). Hopefully that paper will be published in-time to be referenced but this may be beyond the authors control.	We thank the reviewer for this suggestion. The micro-costing development and feasibility study has now been published and is referenced accordingly.
7. Was patient-level clustering taken into account – the variation in the number of visits per patient? For example, identifying the mean per patient, and then using these means to calculate the overall mean. I do not think this is essential, but it could be helpful if there is a small group of patients with a very high number of visits (because raw mean would over-represent these patients).	See answer to comment number 11.
Results<br>8. Related to the above comment, I suggest adding more details about the distribution of visits, given that this is the core unit of analysis. The mean and SD is reported, but	We thank the reviewer for this helpful suggestion. As recommended, we have added the median and range for the number of visits per patient in Table 1 and included the median value in the Results section to provide additional

was this normally distributed? The median, potentially min/max, could be reported in-text. This would help readers to interpret the findings.	information on the distribution of visits. Because the tariff estimates are based on empirically observed mean resource-use inputs per visit, the estimation itself does not rely on distributional assumptions regarding the number of visits per patient. The additional summary statistics are therefore provided to facilitate interpretation of the observed variation in visit intensity across patients.
9. Generally the results are very clear. I checked tables, statistics, and found no inconsistencies or ambiguities. The figures are also well-presented.	We thank the reviewer for this positive assessment of the Results section and figures.
Discussion:<br>10. The authors discuss the omission of medication costs in the limitations section. I agree that this decision likely strengthens the reliability of the findings, given the high heterogeneity in this type of cost. However, for readers unfamiliar with this type of study, it may be helpful to also state that this is a common exclusion in micro-costing studies.	We thank the reviewer for this helpful suggestion. We have revised the limitations section to clarify that the exclusion of medication costs reflects the structural scope of the present activity-based tariff framework. As the objective was to establish a generalisable visit-level tariff, highly variable and diagnosis-specific inputs such as medication costs were modelled separately in order to preserve transparency and comparability across patient groups. We now state this rationale explicitly in the manuscript and clarify that medication costs can be incorporated separately in subsequent economic evaluations (p. 20).
11. The limitation statement regarding repeated measures answers my earlier query regarding whether these were taken into account in the calculations. The justification is reasonable, but adding more details of the distribution of visits would help readers to understand the impact of this. The mean and SD of visits is provided in Table 1, but adding the median and min/max range (or even a histogram in a supplement) would help to understand if outliers with extremely high visit counts had much impact or not.	We thank the reviewer for raising the issue of patient-level clustering and the distribution of visits. The unit of analysis for tariff development was the individual visit, as the objective was to estimate the expected cost per visit rather than the mean cost per patient. Patient-level clustering was therefore not applied in the tariff calculations.<br>To enhance transparency regarding variation in visit intensity, we have now added median and range for total visits per patient, as well as for bed days and visit counts, in Table 1. The median number of total visits per patient was 9 (range 2–27), indicating moderate variation across patients.

	During this revision, we identified and corrected a minor inconsistency in the previously reported mean number of bed days. The corrected value is now presented in Table 1. This correction does not affect the tariff calculations or study conclusions.

Reviewer: 2
Dr. Gudrun Bjørnelv, Norwegian University of Science and Technology
Comments to the Author:
Thank you for giving me the opportunity to read this study where the authors have used a time-driven activity-based micro-costing approach to estimate a hospital-at-home tariff in Denmark. Overall, this is a well-conducted and interesting study. Getting better estimates of the costs in the home- and community based care setting is very important now and in the future when municipalities will bear more of the burden of the aging population. High-quality cost-effectiveness studies will then be warranted, and in these, having good methods to estimate costs also in other settings that in the hospital is important. This study is a timely and warranted contribution to enable this.
I primarily have minor comments that I think would increase the value of the study. Generally, the manuscript is well written and methods seem sound. I think you could get a bit more out of the study by i.e., making a prediction model based on the numbers in the study. This could be a flexible tool to estimate costs of different types of visits (depending on which input variables you ‘turn up or down’ (see comment below). The papers weakest part is the discussion section, where I think the authors can go more in-depth of their own methods, analyses and findings compared to the broader literature to better contextualize the findings.

Comment	*Response*
1. TDABC is several places written out as Trime-Driven Activity-Based Costing with the abbreviation in parentheses – please write the full definition only the first time it is mentioned and use the abbreviation thereafter.	We thank the reviewer for this observation. The full term “Time-Driven Activity-Based Costing (TDABC)” is now defined at first mention only, and the abbreviation TDABC is used consistently throughout the manuscript thereafter.
2. To be able to have a hospital-at-home, I suppose there is a need for a certain capacity that can react if acute episodes occur. This means that persons will have to be on call, but without actually providing care. It was not clear how you distributed this time to the treatments?	We thank the reviewer for raising this important point. In the present setting, Hospital at Home was delivered within the framework of the already established municipal acute-care function and existing hospital emergency services. The capacity to respond to acute episodes, including on-call availability, was therefore embedded in the existing organisational structure and staff remuneration. No additional standby or on-call capacity was established specifically for the study. Consequently, these costs are implicitly reflected

	in the salary-based capacity cost rates and were not allocated as a separate cost component.<br><br>We have added a clarifying statement under Step 5 (p. 8): *“The HaH service was delivered within existing municipal and hospital emergency structures, and no additional standby capacity was established specifically for the study. On-call availability was therefore considered embedded within the salary-based capacity cost rates.”*
3. For the point estimates of the tariff (i.e., the cost per fist (€237.67) and following (€98,30) visit) – why is there no uncertainty in the estimates? I would have expected to see for example 95% confidence intervals surrounding the point estimates? This is the case for all point estimates. If I understand the methods correct, you get these point-estimates based on the equations (1 and 2 depending on whether it is the visits or transport costs you estimate). Using bootstrapping would enable you to make estimates of uncertainties (confidence bounds).	We thank the reviewer for this important comment. We agree that the original version did not quantify sampling uncertainty around the tariff estimates, and this has now been addressed.<br>The tariffs are deterministic functions of empirically observed mean resource-use inputs (time use and activity frequencies) combined with fixed unit-cost parameters, as specified in Equations (1) and (2). To quantify uncertainty arising from sampling variability in the observed resource-use data, we propagated this uncertainty through the tariff equations using Monte Carlo simulation.<br>For each resource-use input included in the model, we derived the mean and corresponding standard error from the underlying dataset. We then performed 10,000 Monte Carlo iterations in which resource-use inputs were sampled from truncated normal distributions (lower bound at zero) centered on the observed means with their respective standard errors. In each iteration, the full tariff model was recalculated, yielding empirical distributions for the tariff components and total tariffs. Percentile-based 95% confidence intervals were derived from the 2.5th and 97.5th percentiles of these simulated distributions.<br>We chose Monte Carlo propagation rather than non-parametric bootstrapping because the tariff is constructed as a deterministic function of mean resource-use inputs rather than estimated through a regression framework. In this context, uncertainty arises from sampling variability in the mean inputs, and Monte Carlo simulation

	provides a transparent method to propagate this variability through the cost equations.<br>Unit-cost parameters were held constant in the Monte Carlo analysis, as these reflect parameter assumptions rather than sampling variability in observed resource use. Their influence is explored separately in deterministic sensitivity analyses.<br>During the implementation of the uncertainty analysis, minor inconsistencies in the resource-use input tables were identified and corrected, resulting in small revisions to the reported point estimates. These corrections do not affect the overall conclusions but improve internal consistency and transparency. The Methods and Results sections have been revised accordingly.
4. In the text (result section), although you have a lot of the numbers in the tables, I think the read would be better if you also have some numbers in the text. For example, "First visits most frequently occurred on weekday evenings (50%) and days (33%)…etc).	We thank the reviewer for this suggestion. We have added additional key numerical values for the main cost components directly in the Results text, including point estimates and 95% confidence intervals for staff, equipment and transport components, as well as selected values from the sensitivity analyses, to improve transparency and readability.
5. It would be interesting to see how different patients conditions influence the cost-estimates (i.e., are the costs different depending on whether the patient has pneumonia, acute cellulitis, UTI or other diagnoses?)	We thank the reviewer for this thoughtful suggestion. We agree that exploring potential cost differences across diagnostic groups would be of interest, as underlying clinical conditions may influence visit intensity and resource use. However, the objective of the present study was to develop a general activity-based tariff framework reflecting observed mean resource use per visit across the study population, irrespective of diagnosis. The tariff is therefore constructed as a diagnosis-independent estimate of expected cost per visit. Diagnosis-specific cost analyses would require a different analytical framework and were considered beyond the scope of the present study.<br><br>We have now stated this in the limitations p. 17: *"Although we did not conduct analyses to assess the impact of repeated measures, uncertainty and robustness were addressed through sensitivity analyses of key cost drivers. Similarly, while*

	differences in resource use across diagnostic groups may be clinically relevant, the present tariff was constructed as a diagnosis-independent activity-based estimate and does not capture diagnosis-specific cost variation."
6. For table 2, I think it would be interesting with more information on the different cost components that make up the finale estimates. Could you make a table where you show the average resource use (i.e., time spent for different professions) and unit costs? Then the reader/ user can estimate the total costs themselves by timing the average with the unit cost. This would make the work much more transparent! Now there is a 'black box' since you do not indicate actual average resource use and unit costs that go into the equations 1 and 2.	We thank the reviewer for this important comment. To enhance transparency and address the concern regarding the "black box" character of the tariff calculations, we have implemented several revisions.<br><br>First, Table 2 has been expanded to provide a clearer breakdown of cost components across time periods. Second, Step 8 in the Methods section and Equations (1) and (2) have been revised to more explicitly reflect the actual calculation procedure, including the weighting of staff-related costs for follow-up visits and the separate addition of activity-based cost components.<br><br>In addition, we have added supplementary material presenting the underlying input parameters used in the tariff calculations. Supplementary file 2 reports the capacity cost rates (unit costs) for all relevant staff categories. Supplementary file 4 presents the mean staff time inputs and activity frequencies by visit type and time period. Finally, Supplementary file 5 provides a detailed worked example (follow-up visit, weekday daytime) demonstrating how total treatment cost per visit is reconstructed from mean resource use and unit costs.<br><br>We believe these additions substantially improve transparency and enable readers to reconstruct the tariff estimates directly from the reported inputs.
7. Also, aligned with the question above – it should be possible for the reader to understand the numbers going into the equations 1 and 2. So: could you make a prediction model where readers/ users of the work can vary different factors in the	We thank the reviewer for this constructive suggestion. The primary objective of the present study was to establish a transparent, empirically grounded activity-based tariff framework rather than to develop an interactive prediction interface. However, the tariff model is fully

equation up and down, and see how this influences the findings? Make a flexible prediction model of the cost per visit? For example, what happens if the time spent by nurses increases or decreases? Or if their salary increases/ decreases? This would also enable people to adjust both the unit and the unit costs according to their setting (i.e., if using your model in another country such as Sweden or Norway).	parameterised and explicitly equation-based. All underlying input parameters, including mean staff time by category, activity frequencies, capacity cost rates and overhead assumptions, are now reported in the Supplementary material together with the formal tariff equations.<br>This structure allows users to substitute alternative values for time inputs, salary levels, overhead rates or activity frequencies and directly recalculate the resulting tariff estimates within their own organisational or national context. In this sense, the model functions as a generalisable costing framework that can be recalibrated to reflect local assumptions.<br>In addition, the deterministic sensitivity analyses presented in the manuscript illustrate the quantitative impact of varying key parameters, thereby demonstrating the model's responsiveness to changes in structural cost drivers. We therefore believe that the revised presentation provides sufficient transparency and flexibility to support adaptation and contextual recalibration without extending the scope of the present study to include development of a separate prediction tool.
8. In Table 4, I do not understand the headings (e.g., why does it for the overhead costs say 0%/0%, 10%/18% and 15%/25%?). I understand it is low, base case and high, but why are there two numbers for low/ high?	We thank the reviewer for pointing this out. The two percentages reflect different overhead assumptions applied to municipal and regional staff, respectively. We have clarified this directly below Table 4 to improve transparency (p. 16).
9. When you show the application of the tariff – can you include the actual calculations (so how do you come to €2,386.67, €2,590.97 and € 4,225.37? And, are the decimal numbers necessary?	We thank the reviewer for this helpful comment. We have revised the section illustrating the application of the tariff to explicitly show the underlying calculations. The number of first and following visits is now stated for each example, and the total cost is presented as the product of the respective tariffs and visit counts (e.g., 1 × first-visit tariff + 14 × following-visit tariff).<br><br>In addition, for improved readability, the illustrative totals have been rounded to the nearest euro, and this rounding approach is stated in the text. The underlying tariff estimates

	remain reported with two decimals in the main tables to ensure consistency and transparency (p. 16-17).
10. In the discussion you start by saying that you developed and validated this – I see you have developed it using sound methods. But how was it validated?	We thank the reviewer for this important observation. We agree that the use of the term "validated" was not appropriate, as no formal validation of the tariff model was conducted. The model was developed using prospectively collected resource-use data and predefined costing assumptions, but it was not externally validated. We have therefore revised the wording in the Discussion to remove this term and ensure that the description of the study is precise and accurate.
11. Overall, the discussion section is weak. The first two pages are completely without any references or reflections related to other literature – and the rest is too weak. I think you should exploit the discussion section to really dig deeper into the comparison to other methods, other findings (what are the estimated costs for HAH in the literature) ++ In hospitals, for example, we use diagnosis-related groups and have methods to estimate costs per DRG. How are the methods used there different/ similar to your methods? Often, cost-effectiveness analyses includes treatment in hospital and in the municipal setting – if methods used to estimate costs in the hospital differs greatly from those used to estimate costs in the municipality this might be problematic. This should be elaborated.	We thank the reviewer for this constructive and important comment. In response, we have substantially revised and expanded the Discussion section to provide a deeper methodological comparison and a more detailed engagement with the existing literature.<br><br>The revised section *Methodological considerations*, (p. 17-18) now elaborates explicitly on the conceptual and methodological differences between DRG-based hospital reimbursement and activity-level costing using TDABC. We clarify that DRG tariffs are derived from aggregated expenditure data and represent average costs per admission, whereas the present study applies bottom-up activity-based measurement linked to capacity cost rates. The revised text further discusses the distinction between average reimbursement tariffs and marginal or opportunity costs in economic evaluation, and the implications of combining different costing paradigms within cross-sectoral analyses.<br><br>In addition, the section *Comparison with existing evidence* (p. 18-19) has been expanded to provide a more detailed discussion of published HaH cost findings. We now describe the general tendency towards reported cost savings while also addressing heterogeneity in costing perspective,

	time horizon, and inclusion of cost components. Importantly, we clarify that most published studies report episode-level costs, whereas the present study develops a visit-level tariff, and that these costing objects are not directly comparable. We believe these revisions address the reviewer's request for a more in-depth methodological and comparative discussion.
12. Also, I would like to have a broader discussion on the applicability of the methods to other municipal interventions – how should this be applied, updated and rolled out to enable municipalities to have better control over the costs of the care they provide?	We appreciate this important suggestion and have expanded the section *Implications for policy and practice* (p. 20-21) to address the broader applicability of the framework.<br><br>The revised text now discusses how the TDABC-based tariff structure may be applicable to other decentralised and staff-intensive municipal health services beyond HaH. We elaborate on the practical requirements for broader implementation, including systematic process mapping, definition of standardised care activities, and periodic updating of capacity cost rates to reflect organisational and wage changes. We further clarify that activity-based costing can function not only as a tariff development tool but also as an ongoing management instrument supporting financial oversight and cost transparency in municipal care settings. These additions respond directly to the reviewer's request for a broader discussion of implementation and scalability.
13. More elaborate discussion (except for what I suggest) is also warranted.	In response to this general comment, we have comprehensively revised the Discussion section to increase its analytical depth and coherence. The revised manuscript now includes an expanded methodological comparison between DRG-based hospital costing and activity-level municipal costing, a more detailed engagement with the heterogeneity of cost findings in the HaH literature, and an extended discussion of applicability and implementation in municipal contexts. We believe that these revisions substantially strengthen the Discussion and

	provide a clearer interpretation of the study's methodological and policy implications.
14. Also, I do not see any information of the ethics in the study, this should be included before the result section.	We thank the reviewer for pointing this out. The information regarding ethical approval has now been added to the Methods section under a dedicated subsection before the Results section.
